# Understanding the mental health of doctoral researchers: a mixed methods systematic review with meta-analysis and meta-synthesis

**DOI:** 10.1186/s13643-020-01443-1

**Published:** 2020-08-26

**Authors:** Cassie M. Hazell, Laura Chapman, Sophie F. Valeix, Paul Roberts, Jeremy E. Niven, Clio Berry

**Affiliations:** 1 School of Social Sciences, University of Westminster, 115 New Cavendish Street, London, W1W 6UW UK; 2 School of Psychology, University of Sussex, Falmer, Brighton, BN1 9QJ UK; 3 Research and Enterprise, University of Sussex, Falmer, Brighton, BN1 9RH UK; 4 Centre for Higher Education and Equity Research, University of Sussex, Falmer, Brighton, BN1 9RH UK; 5 School of Life Sciences, University of Sussex, Falmer, Brighton, BN1 9QG UK; 6 Primary Care and Public Health, Brighton and Sussex Medical School and School of Psychology, University of Sussex, Falmer, Brighton, BN1 9PH UK

**Keywords:** Mental health, PhD students, doctoral researchers, Postgraduate researchers, Systematic review

## Abstract

**Background:**

Data from studies with undergraduate and postgraduate taught students suggest that they are at an increased risk of having mental health problems, compared to the general population. By contrast, the literature on doctoral researchers (DRs) is far more disparate and unclear. There is a need to bring together current findings and identify what questions still need to be answered.

**Methods:**

We conducted a mixed methods systematic review to summarise the research on doctoral researchers’ (DRs) mental health. Our search revealed 52 articles that were included in this review.

**Results:**

The results of our meta-analysis found that DRs reported significantly higher stress levels compared with population norm data. Using meta-analyses and meta-synthesis techniques, we found the risk factors with the strongest evidence base were isolation and identifying as female. Social support, viewing the PhD as a process, a positive student-supervisor relationship and engaging in self-care were the most well-established protective factors.

**Conclusions:**

We have identified a critical need for researchers to better coordinate data collection to aid future reviews and allow for clinically meaningful conclusions to be drawn.

**Systematic review registration:**

PROSPERO registration CRD42018092867

## Background

Student mental health has become a regular feature across media outlets in the United Kingdom (UK), with frequent warnings in the media that the sector is facing a ‘mental health crisis’ [1]. These claims are largely based on the work of regulatory authorities and ‘grey’ literature. Such sources corroborate an increase in the prevalence of mental health difficulties amongst students. In 2013, 1 in 5 students reported having a mental health problem [2]. Only 3 years later, however, this figure increased to 1 in 4 [3]. In real terms, this equates to 21,435 students disclosing mental health problems in 2013 rising to 49,265 in 2017 [4]. Data from the Higher Education Statistics Agency (HESA) demonstrates a 210% increase in the number of students terminating their studies reportedly due to poor mental health [5], while the number of students dying by suicide has consistently increased in the past decade [6].

This issue is not isolated to the UK. In the United States (US), the prevalence of student mental health problems and use of counselling services has steadily risen over the past 6 years [7]. A large international survey of more than 14,000 students across 8 countries (Australia, Belgium, Germany, Mexico, Northern Ireland, South Africa, Spain and the United States) found that 35% of students met the diagnostic criteria for at least one common mental health condition, with highest rates found in Australia and Germany [8].

The above figures all pertain to undergraduate students. Finding equivalent information for postgraduate students is more difficult, and where available tends to combine data for postgraduate taught students and doctoral researchers (DRs; also known as PhD students or postgraduate researchers) (e.g. [4]). The latest trend analysis based on data from 36 countries suggests that approximately 2.3% of people will enrol in a PhD programme during their lifetime [9]. The countries with the highest number of DRs are the US, Germany and the UK [10]. At present, there are more than 281,360 DRs currently registered across these three countries alone [11, 12], making them a significant part of the university population. The aim of this systematic review is to bring attention specifically to the mental health of DRs by summarising the available evidence on this issue.

Using a mixed methods approach, including meta-analysis and meta-synthesis, this review seeks to answer three research questions: (1) What is the prevalence of mental health difficulties amongst DRs? (2) What are the risk factors associated with poor mental health in DRs? And (3) what are the protective factors associated with good mental health in DRs?

## Methods

### Literature search

We conducted a search of the titles and abstracts of all article types within the following databases: AMED, BNI, CINAHL, Embase, HBE, HMIC, Medline, PsycInfo, PubMed, Scopus and Web of Science. The same search terms were used within all of the databases, and the search was completed on the 13th April 2018. Our search terms were selected to capture the variable terms used to describe DRs, as well as the terms used to describe mental health, mental health problems and related constructs. We also reviewed the reference lists of all the papers included in this review. Full details of the search strategy are provided in the supplementary material.

### Inclusion criteria

Articles meeting the following criteria were considered eligible for inclusion: (1) the full text was available in English; (2) the article presented empirical data; (3) all study participants, or a clearly delineated sub-set, were studying at the doctoral level for a research degree (DRs or equivalent); and (4) the data collected related to mental health constructs. The last of these criteria was operationalised (a) for quantitative studies as having at least one mental health-related outcome measure, and (b) for qualitative studies as having a discussion guide that included questions related to mental health. We included university-published theses and dissertations as these are subjected to a minimum level of peer-review by examiners.

### Exclusion criteria

In order to reduce heterogeneity and focus the review on doctoral research as opposed to practice-based training, we excluded articles where participants were studying at the doctoral level, but their training did not focus on research (e.g. PsyD doctorate in Clinical Psychology).

### Screening articles

**Fig. 1:**
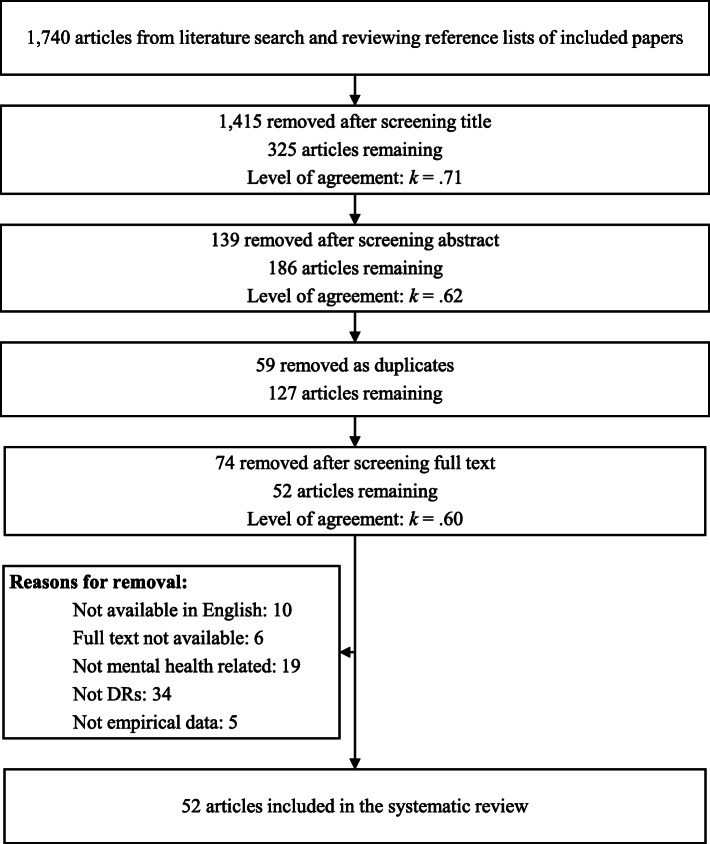
PRISMA diagram of literature review process

Papers were screened by one of the present authors at the level of title, then abstract, and finally at full text (Fig. 1). Duplicates were removed after screening at abstract. At each level of screening, a random 20% sub-set of articles were double screened by another author, and levels of agreement were calculated (Cohen’s kappa [13]). Where disagreements occurred between authors, a third author was consulted to decide whether the paper should or should not be included. All kappa values evidence at least moderate agreement between authors [14]—see Fig. 1 for exact kappa values.

### Data extraction

This review reports on both quantitative and qualitative findings, and separate extraction methods were used for each. Data extraction was performed by authors CH, CB, SV and LC.

#### Quantitative data extraction

The articles in this review used varying methods and measures. To accommodate this heterogeneity, multiple approaches were used to extract quantitative data. Where available, we extracted (a) descriptive statistics, (b) correlations and (c) a list of key findings. For all mental health outcome measures, we extracted the means and standard deviations for the DR participants, and where available for the control group (descriptive statistics). For studies utilising a within-subjects study design, we extracted data where a mental health outcome measure was correlated with another construct (correlations). Finally, to ensure that we did not lose important findings that did not use descriptive statistics or correlations, we extracted the key findings from the results sections of each paper (list of key findings). Key findings were identified as any type of statistical analysis that included at least one mental health outcome.

#### Qualitative data extraction

In line with the meta-ethnographic method [15] and our interest in the empirical data as well as the authors’ interpretations thereof, i.e. the findings of each article [16], the data extracted from the articles comprised both results/findings and discussion/conclusion sections. For articles reporting qualitative findings, we extracted the results and discussion sections from articles verbatim. Where articles used mixed methods, only the qualitative section of the results was extracted. Methodological and setting details from each article were also extracted and provided (see Appendix A) in order to contextualise the studies.

### Data analysis

#### Quantitative data analysis

##### Descriptive statistics

We present frequencies and percentages of the constructs measured, the tools used and whether basic descriptive statistics (*M* and *SD*) were reported. The full data file is available from the first author upon request.

##### Effect sizes

Where studies had a control group, we calculated a between-group effect size (Cohen’s *d*) using the formula reported by Wilson [17], and interpreted using the standard criteria [13]. For all other studies, we sought to compare results with normative data where the following criteria were satisfied: (a) at least three studies reported data using the same mental health assessment tool; (b) empirical normative data were available; and (c) the scale mean/total had been calculated following original authors’ instructions. Only the Perceived Stress Scale (PSS) 10- [18] and 14-item versions [19] met these criteria. Normative data were available from a sample of adults living in the United States: collected in 2009 for the 10-item version (*n* = 2000; *M* = 15.21; *SD* = 7.28) [20] and in 1983 for the 14-item version (*n* = 2355; *M* = 19.62; *SD* = 7.49) [18].

The meta-analysis of PSS data was conducted using MedCalc [21], and based on a random effects model, as recommended by [22]. The between-group effect sizes (DRs versus US norms) were calculated comparing PSS means and standard deviations in the respective groups. The effect sizes were weighted using the variable variances [23].

##### Correlations

Where at least three studies reported data reflecting a bivariate association between a mental health and another variable, we summarised this data into a meta-analysis using the reported *r* coefficients and sample sizes. Again, we used MedCalc [21] to conduct the analysis using a random effects model, based on the procedure outlined by Borenstein, Hedges, Higgins and Rothstein [24]. This analysis approach involves converting correlation coefficients into Fisher’s *z* values [25], calculating the summary of Fisher’s *z*, and then converting this to a summary correlation coefficient (*r*). The effect sizes were weighted in line with the Hedges and Ollkin [23] method. Heterogeneity was assessed using the *Q* statistic, and *I*^2^ value—both were interpreted according to the GRADE criteria [26]. Where correlations could not be summarised within a meta-analysis, we have reported these descriptively.

##### Narration

Due to the heterogenous nature of the studies, the above methods could not capture all of the quantitative data. Therefore, additional data (e.g. frequencies, statistical tests) reported in the identified articles was collated into a single document, coded as relating to prevalence, risk or protective factors and reported as a narrative review.

#### Qualitative data analysis

##### Coding

We used thematic analytic methods to analyse the qualitative data. We followed the thematic synthesis method [16, 27] and were informed by a thematic analysis approach [28, 29]. We took a critical realist epistemological stance [30, 31] and aimed to bring together an analysis reflecting meaningful patterns amongst the data [29] or demi-regularities, and identifying potential social mechanisms that might influence the experience of such phenomena [31]. The focus of the meta-synthesis is interpretative rather than aggregative [32].

Coding was line by line, open and complete. Following line-by-line coding of all articles, a thematic map was created. Codes were entered on an article-by-article basis and then grouped and re-grouped into meaningful patterns. Comparisons were made across studies to attempt to identify demi-regularities or patterns and contradictions or points of departure. The thematic map was reviewed in consultation with other authors to inductively create and refine themes. Thematic summaries were created and brought together into a first draft of the thematic structure. At this point, each theme was compared against the line-by-line codes and the original articles in order to check its fit and to populate the written account with illustrative quotations.

##### Research rigour

The qualitative analysis was informed by independent coding by authors CB and SV, and analytic discussions with CH, SV and LC. Our objective was not to capture or achieve inter-rater reliability, rather the analysis was strengthened through involvement of authors from diverse backgrounds including past and recent PhD completion, experiences of mental health problems during PhD completion, PhD supervision experience, experience as employees in a UK university doctoral school and different nationalities. In order to enhance reflexivity, CB used a journal throughout the analytic process to help notice and bracket personal reflections on the data and the ways in which these personal reflections might impact on the interpretation [29, 33]. The ENTREQ checklist [34] was consulted in the preparation of this report to improve the quality of reporting.

#### Quality assessment

##### Quantitative data

The quality of the quantitative papers was assessed using the STROBE combined checklist [35]. A random 20% sub-sample of these studies were double-coded and inter-rater agreement was 0.70, indicating ‘substantial’ agreement [14]. The maximum possible quality score was 23, with a higher score indicating greater quality, with the mean average of 15.97, and a range from 0 to 22. The most frequently low-scoring criteria were incomplete reporting regarding the management of missing data, and lack of reported efforts to address potential causes of bias.

##### Qualitative data

There appeared to be no discernible pattern in the perceived quality of studies; the highest [36–40] and lowest scoring [41–46] studies reflected both theses and journal publications, a variety of locations and settings and different methodologies. The most frequent low-scoring criteria were relating to the authors’ positions and reflections thereof (i.e. ‘Qualitative approach and research paradigm’, ‘Researcher characteristics and reflexivity’, ‘Techniques to enhance trustworthiness’, ‘Limitations’, ‘Conflict of interest and Funding’). Discussions of ethical issues and approval processes was also frequently absent. We identified that we foregrounded higher quality studies in our synthesis in that these studies appeared to have greater contributions reflected in the shape and content of the themes developed and were more likely to be the sources of the selected illustrative quotes.

#### Mixed methods approach

The goal of this review is to answer the review questions by synthesising the findings from both quantitative and/or qualitative studies. To achieve our goal, we adopted an integrated approach [47], whereby we used both quantitative and qualitative methods to answer the same review question, and draw a synthesised conclusion. Different analysis approaches were used for the quantitative and qualitative data and are therefore initially reported separately within the methods. A separate synthesised summary of the findings is then provided.

## Results

### Overview of literature

Of the 52 papers included in this review (Table 1), 7 were qualitative, 29 were quantitative and 16 mixed methods. Most articles (35) were peer-reviewed papers, and the minority were theses (17). Only four of the articles included a control group; in three instances comprising students (but not DRs) and in the other drawn from the general population.

**Table 1:** List of studies included in this review

			Sample size					Scale		
Article	Article type	Method	Total PhD *n*	Male *n*	Female *n*	Other *n*	Age *M*(*SD*)	Universities	Countries	Lead country
Acker et al. (2015) [36]	P	Qualitative	27	5	22	0	-(−)	Single	Single	Canada
Appel et al. (2003) [37]	P	Mixed	159	77	82	0	-(−)	Single	Single	Sweden
Bauer (2016) [48]	D	Quantitative	118	28	90	0	-(−)	Multiple	Single	USA
Bazrafkan et al. (2016) [41]	P	Qualitative	–	–	–	–	32(9)	Multiple	Single	Iran
Begun et al. (2017) [49]	P	Mixed	281	219	60	2	38(9)	Multiple	Single	USA
Benjamin et al. (2017) [50]	P	Qualitative	29	11	18	0	-(−)	Multiple	Single	USA
Benesek (1998) [51]	D	Quantitative	66	11	55	0	-(−)	Multiple	Single	USA
Bireda (2015) [52]	P	Qualitative	5	0	5	0	-(−)	Single	Single	Ethiopia
Bolliger et al. (2012) [53]	P	Quantitative	84	-	-	-	42(−)	Single	Single	USA
Cole (2008) [54]	D	Quantitative	261	98	161	0	-(−)	Multiple	Multiple	-
Cotterall (2013) [42]	P	Qualitative	6	3	3	0	-(−)	Single	Single	Australia
Devine et al. (2017) [55]	P	Mixed*	183	33	150	0	33(6)	Multiple	Multiple	Canada
Devonport et al. (2014) [38]	P	Qualitative	2	2	0	0	27(−)	Single	Single	UK
Drake (2010) [56]	D	Quantitative	220	40	180	0	28(5)	Multiple	Single	USA
Dumitrescu (2016) [57]	D	Quantitative	153	37	115	1	34(8)	Multiple	Single	USA
El-Ghoroury et al. (2012) [58]	P	Quantitative	209	–	–	–	-(−)	Multiple	Single	USA
Enzor (2017) [39]	D	Qualitative	10	2	8	0	-(−)	Multiple	Single	-
Haynes et al. (2012) [59]	P	Qualitative	8	0	8	0	-(−)	Single	Single	USA
Hill (2010) [60]	D	Quantitative	64	29	35	0	-(−)	Single	Single	USA
Holahan (1979) [61]	P	Quantitative	377	0	377	0	-(−)	Single	Single	USA
Hunter et al. (2016) [62]	P	Mixed*	186	150	36	0	33(7)	Multiple	Multiple	Canada
Kaufman (2004) [43]	D	Mixed	41	7	33	0	41(−)	Single	Single	USA
Kaufman (2006) [63]	P	Quantitative	41	7	34	0	41(−)	Single	Single	USA
Kenty (2000) [44]	P	Mixed	111	0	111	0	45(43)	Multiple	Single	USA
Kurtz-Costes et al. (2006) [40]	P	Qualitative	20	8	12	0	-(−)	Single	Single	USA
Levecque et al. (2017) [64]	P	Quantitative	3659	–	–	–	28(5)	Multiple	Single	Finland
Lonka et al. (2014) [65]	P	Quantitative	669	168	496	0	39(-)	Single	Single	Finland
Lowe (2015) [66]	D	Quantitative	338	60	276	2	-(-)	Multiple	Single	USA
Marais et al. (2018) [67]	P	Quantitative	136	36	100	0	-(−)	Single	Single	France
Martinez et al. (2013) [68]	P	Qualitative	5	–	–	–	-(−)	Single	Single	USA
McGregor et al. (2008) [69]	P	Quantitative	10	3	7	0	-(−)	Single	Single	USA
Nelson (2014) [70]	D	Quantitative	156	47	109	0	-(−)	Multiple	Single	USA
Nottingham (2017) [71]	D	Quantitative	116	40	76	0	-(−)	Multiple	Single	USA
Orozco (2014) [72]	D	Quantitative	161	23	138	0	-(−)	Single	Single	USA
Peters (2007) [73]	D	Quantitative	104	24	79	0	-(−)	Single	Single	USA
Pifer et al. (2014) [74]	P	Qualitative	31	17	14	0	33(−)	Single	Single	USA
Platt et al. (1995) [75]	P	Quantitative	53	–	–	–	-(−)	Single	Single	USA
Pychyl (1995) [76]	D	Mixed*	19	8	11	0	-(−)	Single	Single	Canada
Pychyl et al. (1998) [77]	P	Mixed	81	36	45	0	35(−)	Single	Single	Canada
Rocha-Singh (1994) [78]	P	Quantitative	469	178	291	0	-(−)	Single	Single	USA
Scheidler (2008) [79]	D	Quantitative	507	141	363	3	42(−)	Multiple	Single	USA
Scrubb (1997) [45]	D	Mixed	156	80	76	0	-(−)	Single	Single	USA
Sekas et al. (1980) [80]	P	Quantitative	42	–	–	–	-(−)	Single	Single	USA
Stubb et al. (2011) [81]	P	Mixed	669	168	496	0	39(−)	Single	Single	Finland
Stubb et al. (2012) [82]	P	Mixed	669	–	–	–	-(−)	Single	Single	Finland
Ülkü-Steiner et al. (2000) [83]	P	Quantitative	714	293	421	0	-(−)	Single	Single	USA
Usman Yousaf et al. (2016) [46]	P	Qualitative	8	–	–	–	-(−)	Single	Single	Malaysia
Volkert et al. (2018) [84]	P	Quantitative	835	–	–	–	-(−)	Multiple	Single	USA
Waaijer et al. (2016) [85]	P	Quantitative	218	111	107	0	-(−)	Single	Single	Netherlands
Wang et al. (2010) [86]	P	Qualitative	1	1	0	0	30(N/A)	Single	Single	Taiwan
Williams (2014) [87]	D	Quantitative	140	37	101	2	29(6)	Multiple	Single	USA
Wright (2006) [88]	P	Quantitative	15	7	8	0	-(−)	Single	Single	UK

M and SD rounded to whole figures; *D* dissertation, *P* peer reviewed paper, *N*/*A* not applicable, – = not reported, *USA* United Stated of America, *UK* United Kingdom; *Study used mixed methods, but only qualitative data were used in this review as quantitative data did not pertain to mental health

### Quantitative results

#### Descriptive statistics

Thirty-five papers reported quantitative data, providing 52 reported sets of mental health related data (an average of 1.49 measures per study): 24 (68.57%) measured stress, 10 (28.57%) anxiety, 9 (25.71%) general wellbeing, 5 (14.29%) social support, 3 (8.57%) depression and 1 (2.86%) self-esteem. Five studies (9.62%) used an unvalidated scale created for the purposes of the study. Fifteen studies (28.85%) did not report descriptive statistics.

#### Effect sizes

Of the four studies that included a control group, only two of these reported descriptive statistics for both groups on a mental health outcome [66, 69]. There is a small (Cohen’s *d* = 0.27) and large between-group effect (Cohen’s *d* = 1.15) when DRs were compared to undergraduate and postgraduate clinical psychology students respectively in terms of self-reported stress.

**Fig. 2:**
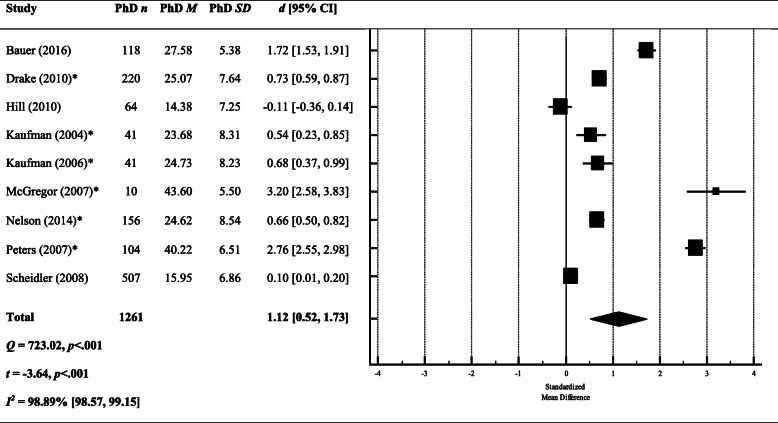
A meta-analysis of between-group effect sizes (Cohen’s *d*) comparing PSS scores (both 10- and 14-item versions) from DRs and normative population data. *Studies using the 14 item version of the PSS; a positive effect size indicates DRs had a higher score on the PSS; a negative effect size indicates that the normative data produced a higher score on the PSS; black diamond = total effect size (based on random effects model); *d* = Cohen’s *d*; *Q* = heterogeneity; *Z* = *z* score; *I*^2^ = proportion of variance due to between-study heterogeneity; *p* = exact *p* value

The meta-analysis of DR scores on the PSS (both 10- and 14-item versions) compared to population normative data produced a large and significant between-group effect size (*d* = 1.12, 95% CI [0.52, 1.73]) in favour of DRs scoring higher on the PSS than the general population (Fig. 2), suggesting DRs experience significantly elevated stress. However, these findings should be interpreted in light of the significant between-study heterogeneity that can be classified as ‘considerable’ [26].

To explore this heterogeneity, we re-ran the meta-analysis separately for the 10- and 14-item versions. The effect size remained large and significant when looking only at the studies using the 14-item version (*k* = 6; *d* = 1.41, 95% CI [0.63, 2.19]), but was reduced and no longer significant when looking at the 10-item version only (*k* = 3; *d* = 0.57, 95% CI [− 0.51, 1.64]). However, both effect sizes were still marred by significant heterogeneity between studies (10-item: *Q* = 232.02, *p* < .001; 14-item: *Q* = 356.76, *p* < .001).

#### Correlations

Studies reported sufficient correlations for two separate meta-analyses; the first assessing the relationship between stress (PSS [18, 19]) and perceived support, and the second between stress (PSS) and academic performance.

##### Stress x support

**Fig. 3:**
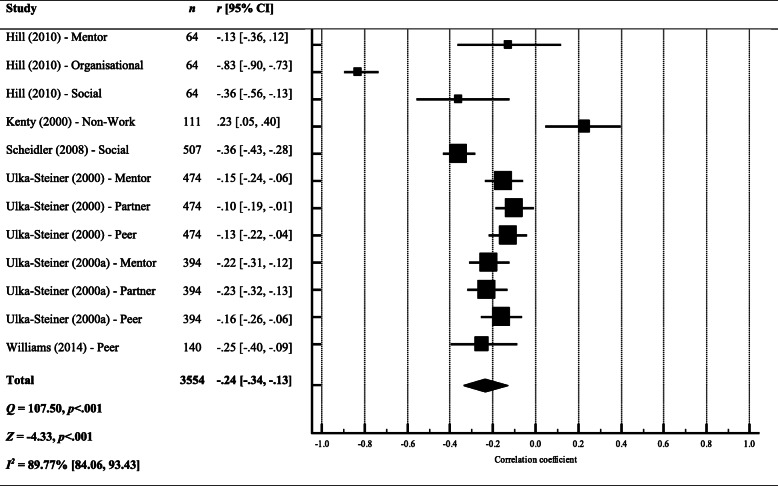
Forest plot and meta-analysis of correlation coefficients testing the relationship between stress and perceived support. Black diamond = total effect size (based on random effects model); *r* = Pearson’s *r*; *Q* = heterogeneity; *Z* = *z* score; *I*^2^ = proportion of variance due to between-study heterogeneity; *p* = exact *p* value

We included all measures related to support irrespective of whom that support came from (e.g. partner support, peer support, mentor support). The overall effect size suggests a small and significant negative correlation between stress and support (*r* = − .24, 95% CI [− 0.34, − 0.13]) (see Fig. 3), meaning that low support is associated with greater perceived stress. However, the results should be interpreted in light of the significant heterogeneity between studies. The *I*^2^ value quantifies this heterogeneity as almost 90% of the variance being explained by between-study heterogeneity, which is classified as ‘substantial’ (26).

##### Stress x performance

**Fig. 4:**
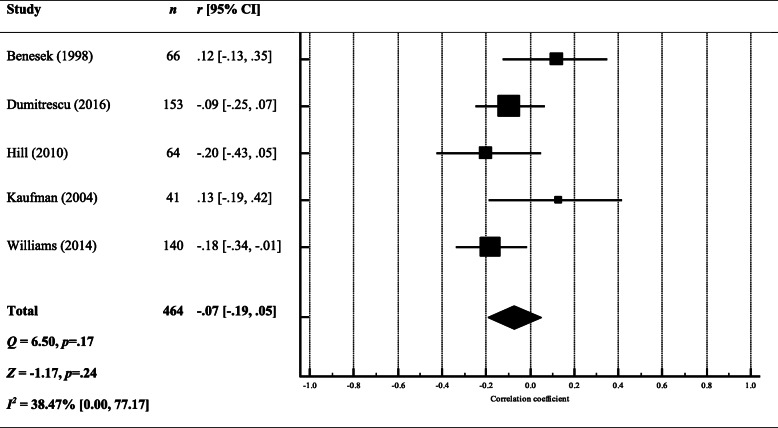
Forest plot and meta-analysis of correlation coefficients testing the relationship between stress and performance. Black diamond = total effect size (based on random effects model); *r* = Pearson’s *r*; *Q* = heterogeneity; *Z* = *z* score; *I*^*2*^ = proportion of variance due to between-study heterogeneity; *p* = exact *p* value

The overall effect size suggests that there is no relationship between stress and performance in their studies (*r* = − .07, 95% CI [− 0.19, 0.05]) (see Fig. 4), meaning that DRs perception of their progress was not associated with their perceived stress This finding suggests that the amount of progress that DRs were making during their studies was not associated with stress levels.

##### Other correlations

**Fig. 5:**
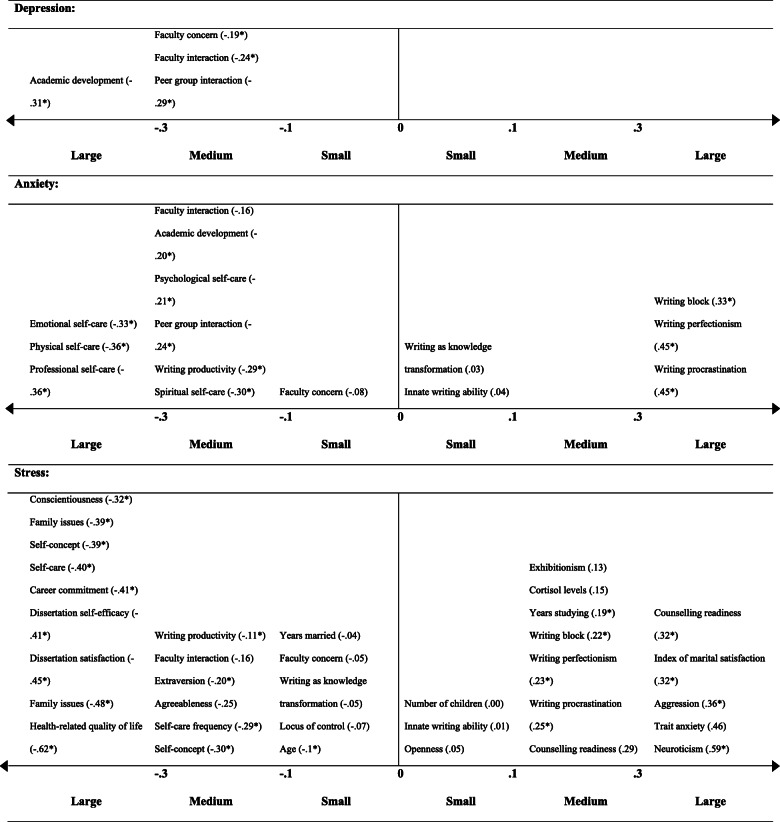
Correlation coefficients testing the relationship between a mental health outcome and other construct. Correlation coefficients are given in brackets (*r*); *p < .05; each correlation coefficient reflects the results from a single study

Correlations reported in less than three studies are summarised in Fig. 5. Again, stress was the most commonly tested mental health variable. Self-care and positive feelings towards the thesis were consistently found to negatively correlate with mental health constructs. Negative writing habits (e.g. perfectionism, blocks and procrastination) were consistently found to positively correlate with mental health constructs. The strongest correlations were found between stress, and health related quality of life (*r* = − .62) or neuroticism (*r* = .59), meaning that lower stress was associated with greater quality of life and reduced neuroticism. The weakest relationships (*r* < .10) were found between mental health outcomes and: faculty concern, writing as knowledge transformation, innate writing ability (stress and anxiety), years married, locus of control, number of children and openness (stress only).

#### Narration

##### Prevalence

Several studies reported DR mental health problem prevalence and this ranged from 36.30% [54] to 55.9% [67]. Using clinical cut-offs, 32% were experiencing a common psychiatric disorder [64]; with another study finding that 53.7% met the questionnaire cut-off criteria for depression, and 41.9% for anxiety [67]. One study compared prevalence amongst DRs and the general population, employees and other higher education students; in all instances, DRs had higher levels of psychological distress (non-clinical), and met criteria for a clinical psychiatric disorder more frequently [64].

##### Risk factors

*Demographics* Two studies reported no significant difference between males and females in terms of reported stress [57, 73], but the majority suggested female DRs report greater clinical [80], and non-clinical problems with their mental health [37, 64, 79, 83, 89].

Several studies explored how mental health difficulties differed in relation to demographic variables other than gender, suggesting that being single or not having children was associated with poorer mental health [64] as was a lower socioeconomic status [71]. One study found that mental health difficulties did not differ depending on DRs’ ethnicity [51], but another found that Black students attending ‘historically Black universities’ were significantly more anxious [87]. The majority of the studies were conducted in the US, but only one study tested for cross-cultural differences: reporting that DRs in France were more psychologically distressed than those studying in the UK [67].

*Work-life balance* Year of study did not appear to be associated with greater subjective stress in a study involving clinical psychology DRs (Platt and Schaefer [75]), although other studies suggested greater stress reported by those in the latter part of their studies [89], who viewed their studies as a burden [81], or had external contracts, i.e. not employed by their university [85]. Regression analyses revealed that a common predictor of poor mental health was uncertainty in DR studies; whether in relation to uncertain funding [64] or uncertain progress [80]. More than two-thirds of DRs reported general academic pressure as a cause of stress, and a lack of time as preventing them from looking after themselves [58]. Being isolated was also a strong predictor of stress [84].

##### Protective factors

DRs who more strongly endorsed all of the five-factor personality traits (openness, conscientiousness, extraversion, agreeableness and neuroticism) [66], self-reported higher academic achievement [40] and viewed their studies as a learning process (rather than a means to an end) [82] reported fewer mental health problems. DRs were able to mitigate poor mental health by engaging in self-care [72], having a supervisor with an inspirational leadership style [64] and building coping strategies [56]. The most frequently reported coping strategy was seeking support from other people [37, 58].

### Qualitative results

#### Meta-synthesis

Four higher-order themes were identified: (1) Always alone in the struggle, (2) Death of personhood, (3) The system is sick and (4) Seeing, being and becoming. The first two themes reflect individual risk/vulnerability factors and the processes implicated in the experience of mental distress, the third represents systemic risk and vulnerability factors and the final theme reflects individual and systemic protective mechanisms and transformative influences. See Table 2 for details of the full thematic structure with illustrative quotes.

**Table 2:** Thematic structure with illustrative quotes

Higher order theme	Subthemes	Sub-theme detail and illustrative quotes
1. Always alone in the struggle		
	Invisible, isolated and abandoned	• Feeling invisible and isolated both within and outside the academic environment; ‘…you can go weeks without talking to anybody, even family’ [39] (p. 86). • Friendships characterised by: ▪ Poor quality; ‘…[rarely extending] beyond self-interest and competitiveness and into a realm of personal exchange and interpersonal connectedness.’ [39] (p. 88). ▪ Competitiveness; ‘They’re all fighting amongst each other, thinking you're getting it a lot better than I am … and the effect is that none of these people are getting very much: they're scraping for crumbs on the table’ [36]. ▪ Too shared a sense of experience; ‘…you share the same fears... they are too close to the problem.’ [39] (p. 78). • Departments perceived as failing to support DR connectedness; ‘…when the department was going to create their web site, and there was a discussion about who should be listed. First, they said that the doctoral students shouldn’t be there because they don’t have permanent positions. It’s sick’ [37] (p. 103). • Inclusion within departments is only fleeting or hollow; ‘…when they need people, then we’re included’ [37] (p. 103). • DRs made sense of their isolation as being about their own ‘differentness’; ‘Most participants identified themselves as different from the norm, in the role of the other, in at least one meaningful way’ [74] (p. 20). • The lack of inclusion reinforces broader messages about the nature of an academic career; ‘This lack of departmental community sent a powerful (unintended?) message that scholarly research is a solitary affair.’ [42] (p. 180). • The sense of academia as isolated may lead DRs to opt out; ‘…this information may allow them to self-select out of a career in academia if they find that the department or faculty environment is not likely to provide the type of support they anticipate wanting during their career.’ [62] (p. 52). • Isolation was also apparent in personal relationships, both social and psychological; ‘I’m very alone compared to my friends and family at home. Nobody knows what I go through, what I experience’ [74] (p. 24.)
	It’s not you, it’s me	• DRs expressed a sense of sole responsibility for their PhD; ‘You just have to do your job alone without anyone to help you.’ [81] (p. 40). • DRs felt responsible for finding ways to capture and sustain their supervisor/s attention; ‘I feel like a cog. I know she doesn’t mean to make me feel that way but she has other advisees who probably share more similar interests and some of them get more of her time.’ [74] (p. 20). • DRs felt that they had to take full responsibility for their personal lives and prevent any personal intrusion into the professional; ‘…Ariunaa recounted a harrowing story of having spent three sleepless days and nights at the hospital with her son following a severe seizure. During that time she was unable to contact her husband or to eat because she had left her phone and wallet at home in her rush to meet the ambulance. Through her tears, Ariunaa explained that she had not told her supervisors this story—“Because that's just my life and I should … manage my life” (Ariunaa, 3, 2161).’ [42] (p. 183). • DRs identified an archetypal DR and felt that they fell short of this ideal; ‘Mia spontaneously declared that she was not an “ideal doctoral student”’ [37] (p. 107), thus were not really for whom a PhD opportunity was intended; ‘…[the PhD is] something for the exceptionally gifted. The informants did not feel they belonged to this group’ [37] (p.100). • DRs felt unsure about their capacity to do a PhD; “… insecurity about the doctoral students’ own capacity…expressed many times through reflections concerning internal and external demands (one’s own demands and those of others). Are my research results good enough? Am I good enough? Can I handle what’s expected of me?’ [37] (p. 107). • DRs felt that their inadequacy was reflected back by supervisors; ‘…I worked hard, and read a lot of papers because of the fear of response of the supervisor… but unfortunately… I didn’t know the answers to his questions—and then he got more and more angry’ [41] (p. 235). • Feeling that supervisors treated DRs as if they were inadequate was framed as a cue to work harder; ‘The only way to lower the anxiety is to get things done’ [76] (p. 79). • DRs appeared to be continuously working toward but never achieving the DR ideal; ‘Say there are sixteen waking hours in a day. I should be working seventeen’ [76] (p. 64). • The actual-ideal self discrepancy was associated with punitive self-talk; ‘It’s happened again. I can’t believe I’ve got myself in this position…. I got quite upset really, feeling like I’ve let everyone down again…I’ve just let things simmer rather than tackle this work as effectively as I could and there’s no-one to blame but myself.’ [38] (p. 128). • Self-castigation appeared to be necessary but not sufficient attempt to motivate oneself to improve; ‘…it’s not enough to punish yourself or feel guilty, when you don’t meet a goal; you should notice the reasons for your failure to attain the goals in future’ [41] (p. 234). • Internalisation of perceived failure culminated in DRs making sense of their current and future performance through a lens of inferiority; ‘[DR]: There are some doctoral students at the department who have really been given a lot. Whenever they‘ve wanted something, they‘ve got it. They‘ve gone on a lot of trips, and after they‘ve defended their thesis, they‘ve been offered posts, posts designed specially for them […] I don’t think they‘re going to do that for me. [Interviewer]: Why not? [DR]: I don’t know. I suppose that once again it has to do with the fact that I think everyone else is much better than I am.’ [37] (p. 104).
2. Death of personhood		
	A sacrifice of personal identity	• Enmeshment of self-identity and PhD; ‘…this program and what I am doing is essentially my life and everything else revolves around me doing this [PhD].’ [59] (p. 10). • Priority placed on PhD activities to the detriment of personal relationships; ‘When we went into our honeymoon cabin so to speak, he [the DR’s husband] carried in bags that were very heavy and he said, “What’s in here?” They were filled with books, and he was quite upset about it. But, you know, you have to.’ [76] (p. 61). • DRs had a sense of never being free of their PhD; ‘…they felt that their research was always hanging over them and giving them a guilty conscience. This can be seen as the negative side of the freedom experienced when doing research…there is no clear dividing line between work and leisure time.’ [37] (p. 107). • Time spent on non-PhD activities, even basic activities of living, was seen as indulgent; ‘A guilt trip about eating, the time it takes to cook your meals, the time it takes to eat’ [37] (p. 77). • Self and PhD identity enmeshment is associated with identify conflict; ‘Priscilla feels “it's almost like having split personality for me” in balancing her spiritual and academic priorities’ [59] (p. 7); ‘…all of my worlds are colliding’ [59] (p. 10). • Friends and relatives provided an uncomfortable reflection of the DR’s changing identity; ‘None of them have Ph.D.s and I don’t want to have that elitism. I certainly don’t want to have that interfere with my friendships.’ [74] (p. 24). DRs engaged in selective pruning of personal relationships as deemed necessary for future in academia; ‘I moved away from the person I had been seeing for almost two years and did not maintain the relationship … definitely a personal sacrifice. … I mean it is a trade-off, being married or having a full time partner because it’s great financially and emotionally when you’re really down in the dumps, but it’s also really bad [if one gets pregnant or has relationship issues] … that puts strain, that can prevent you from doing your work. So, I’ve sacrificed so that I can stay on track and stuff, so everybody’s like ‘Oh, you do so much … you do it so well’. But, I also don’t have a personal life.’ [40] (p. 147). • The DR identity perceived as unclear and confusing; ‘Sometimes I consider myself still as a student when it comes to doing research and sometimes I already perceive to be an expert in my area.’ [81] (p. 41).
	Self as parasitic	• DRs conceived of themselves as hindering or harmful to others; ‘I'm just a hindrance to others’ [81] (p. 40). • Problems within the PhD could lead to a feeling of punishing close others; ‘…it seems that because I haven’t done the work I’m punishing her or that we have to make the decision that we don’t see each other because I’ve got this work to do. I think sometimes there does become an overlap and my mood can kind of transfer from one situation to the next.’ [38] (p. 128). • DRs expressed concern that they had sacrificed own and familial financial solvency; ‘Students’ qualitative responses sometimes indicated concern that their own educational debt load would become an intergenerational burden and that they wrestled with the pros and cons of spending resources now on their own education versus being able to plan for their children’s educational futures’ [49] (p. 171).
	Death of self-agency	• DRs expressed feeling overwhelmed by their thesis, ‘…it is a world of information and we may be engulfed by its huge Tsunami waves if we do not know how to boldly dive through this ocean of information …but AH! Unfortunately, it seems I am engulfed’ [46] (p. 19), which had the power to overwhelm or destroy; ‘As a result, the influences and demands within her doctoral program are “sucking the life outta [her]” or cause “crumbling”’ [59] (p. 9). • DRs expressed a lack of personal power within academia generally; ‘There’s not much of a role to take, if you are nothing but at mercy of others.’ [81] (p. 40). • Powerlessness was specifically evident in relation to supervisors; ‘I think it’s very natural to feel inadequate’ [39] (p. 79). • DRs felt that supervisors did not treat them as holistic people; ‘No one ever asks, “How are you doing,” not even faculty or your advisor. The dialogue with staff is always very academic-focused, like, “How far are you on your paper,” “How much have you gotten done.”’ [39] (p. 94). • DRs reported that their supervisors and other academics prioritised their own will above that of DRs; ‘I was trying to run some analyses in another lab. The professor in charge of that lab was willing to help me out, however he requested collaborative work in exchange (i.e. to be co-author). My supervisor didn’t like the idea and the samples were not analysed… My dissertation director approved my chapter and later removed approval after another member of my committee did not like the chapter. I felt betrayed by his failure to stand up for me.’ [62] (p. 49). • DRs felt they were used as a means of research production; ‘I had an idea for research. I told my supervisor…and we came up with some ways of testing it…He told me he decided to submit the manuscript as the only author…This experience has left me not trusting my supervisor and I will not share research ideas with him again… Incompatible sense of ethics; was tired of being lied to and directed to do unethical things’ [62] (p. 50). • DRs felt that retaining their self-agency might be incompatible with maintaining a positive supervisory relationship; ‘…it wasn't of any point to keep arguing with him you know … when you're arguing with a professor … the truth is you really have a lot to lose … so I just compromised … and then sort of we started developing a relationship … (Jack, 1, 260–286). Jack considered the role his supervisor assigned him (division of labour) inappropriate and face threatening. However, conscious of the power dynamics at work, he chose not to resist. Instead, he lowered his sights (object) and chose to focus on ‘just finishing’ the PhD: I guess there was a lot of ambition, but … you just reach a point where you don't really care anymore what happens, all you need to do is just … try to see if you can have the results and try to finish. (Jack, 2, 902–913)’ [42] (p. 183). • DRs appeared to accept issues in their supervision without challenge in a resigned fashion; ‘Mary’s goal of improving her English was thwarted by her supervisor's decision to communicate only in Chinese, yet her respect for her supervisor prevented her from objecting. …. Ariunaa's decision to suppress her anxiety about her son may have been prompted by observing the ‘care-less’ (Lynch, 2010) culture of the academy which ‘values … competitive … and individualistic practices’ (Bansel, 2011, p. 552). Unfortunately, the ‘culture of silence’ reflected in Jack, Mary, Dev and Ariunaa's responses militates against change occurring in the AS [activity system] of doctoral education. Anecdotal evidence from the researcher's network of (local and international) doctoral students suggests that the tensions experienced by the participants are common, as are their reactions. Their silence may have less to do with culture than power.’ [42] (p. 184). • DRs shared litanies of supervisory issues but appeared to silence themselves from explicitly criticising their supervisors; ‘Emily felt stressed and anxious trying to decide how to sequence her co-authors’ names in a forthcoming article: E: …now my co-authors are giving very different amount of inputs … and one of them is trying to keep the work just between me and him [laughs]. And it's very clear, like—“Let's just work, I know they are saying that, I know they think like that, but let’s just keep it between you and I, we’ll continue just sending it back and forth … Ok? And I know they think differently, you think differently, but now this is what we're going to do.” [laughs] [R: How do you feel about –?] E: [laughs] So that’s the situation. (Emily, 5, 593-629) Emily’s evasive response to the researcher's question may indicate her reluctance to blame her stress on her supervisor's behaviour (Mesquita & Frijda, 1992).’ [42] (p. 182). • DRs expressed a lack of self-agency in being able to curate positive life circumstances generally; ‘…and basically I have no friends. I mean my contact with the rest of my family is gone. And that I regret. I don’t think that’s healthy. And I want to change that, but I can’t… don’t have the energy or the time to do that just now.’ [76] (p. 101). • DRs appeared to feel a lack of self-agency in relation to curating a positive future; ‘…without an overall goal I find it very difficult to motivate myself to work’ [38] (p. 127), which undermined their current self-agency and motivation; ‘…then I think the most stressful thing is to think [that] I am doing all this and … there isn’t any certainty of a reward at the end or of job security at the end.’ [40] (p. 146).
3. The system is sick		
	Most everyone’s mad here	• Some DRs emphasised pervasive impact of their mental health problems; ‘I suffer from depression and it is a hard thing to overcome … it is a negative influence on my research and as an individual because you need to fight with something constantly to feel better. It is something you have to overcome constantly. It is an ongoing battle that is never going to end; it is like a constant obstacle in your career and in your personal life. (Participant 173, Latina female)’ [50] (p. 208-9). • Some DRs provided more implicit examples of experiences of mental distress: ▪ ‘I had never experienced so much stress and anxiety during all my life. All systems of my body were disturbed. I was always crying because, I felt… I’m dying’ [41] (p. 236). ▪ ‘I feel depressed, sometimes I cannot do anything even no feeling to go outside’ [46] (p. 20). ▪ ‘I just think sometimes your bed’s your safe place so the longer I stay in there everything else isn’t that real.’ [38] (p. 130). • DRs described a PhD-specific numbness-hypervigilance response; ‘…a duality in the outcomes of exposure to doctoral-level stressors explaining, “the usual stress coupled with not enough sleep can really take a toll on one’s mental and emotional health;” however, “little stressors no longer weigh me down like they used to.” (Enzor, 2017, p. 85), “…but some students were experiencing debilitating panic attacks when they received emails from their supervisors, things like that.”’ [39] (p. 83). • DRs described a complex nexus of factors that could increase mental health vulnerability; ‘The financial issues, different issues that really, you know, affect your health, that really come together to form like a nexus’ [36] (p. 234). • The PhD itself was described as a crucible for the development of mental health problems; ‘I think a lot of people in my program have dealt with very serious mental health issues and problems that were either because of the program or exacerbated by the program. It got to the point where the faculty had to have a serious conversation with the students because they realized that half the students were in therapy’ [39] (p. 83). • DRs felt lucky if they did not experience mental health problems during the PhD; ‘I can understand how people with a mental health history may struggle in grad school because of its many demands, but I’ve been very lucky. I’ve been able to maintain good mental health’ [39] (p. 80). • Supervisors and the system were seen to promote an expectation of DR suffering; ‘Katie mentioned a “proactive” approach to stress on multiple occasions throughout her interview; unfortunately, however, her portrayal of the prevailing attitudes within the doctoral learning environment was significantly more negative. She explained “you’re going to go into this and it is going to be very hard and nobody is going to support you and nobody really cares... this is the expectation that we are to suffer,” she elaborated, “I can’t think of anyone who hasn’t suffered in some way through the doctoral process.”’ [39] (p. 87). • Academics were perceived as uncaring with regard to the mental toll of doing a PhD; ‘… some of the professors could be more conscientious of the psychological toll it can take on their students…Caroline declared, “especially for a psychology program, they [faculty and staff] should be much more aware of the psychological issues that come with being in such a stressful program”’ [39] (p. 81). • The cycle of indigenousness of mental health problems was maintained by poor mental health literacy and lack of mental health and support provision; ‘Caroline: “I think we are afforded them [mental health services], I just haven’t taken advantage of it, even though I probably should have. Things might have been easier for me had I sought out the services, but I didn’t.” Dana: “I don’t know if we have any programs that specifically focus on mental health within our doctoral program.” … Isabelle: “I’m not sure I’ve ever looked into it [mental health services]. I know we have them but I don’t know how to access them.”’ [39] (p. 93). • DRs felt let down by the system; ‘…people (should not) feel as if they are losing their minds’ [59] (p. 8), feeling that there was widespread denial of DR mental health problems; ‘I just think that, like, doctoral programs in general just create a lot of anxieties in students who probably are thinking faculty could do more to help with that.’ [39] (p. 82). • DRs felt that the systemic encouragement of unhealthy lifestyles was tantamount to abuse; ‘The abuse I experienced is hard to characterize, especially in a survey form. It took me a long time to understand what was going on because its style was so insidious - being told that to survive in this profession one had to sleep 4 hours a night, or give one’s whole life, 12-hour days at full speed to the profession’ [62] (p. 51).
	A performance of optimum suffering	• DRs felt that they had to show the right amount of stress and distress or else be perceived as not taking their PhD seriously enough; ‘Some students felt they had to hide their personal lives from the faculty and other students. Taking evenings off, going to the beach, or spending an evening in a bar would be frowned upon by faculty. One man in a gender-balanced programme said: ‘I wouldn’t ever want to come back after a weekend with a tan or something. … That would be really uncool because it would mean that I was doing something fun over the weekend.’ [40] (p. 147). • DRs felt that they should not present themselves as intellectually inferior: ▪ ‘…you don’t want to say, I have no idea, I don’t know the steps I’m suppose[d] to take, and I know I should know them, so, people don’t say that’ [76] (p. 83). ▪ ‘In some classes, it’s a very competitive atmosphere, right, you don't open your mouth unless you have something absolutely brilliant to say’ [36] (p. 235). • DRs felt that they should broadly avoid showing vulnerability; ‘I do my best to pretend [my illness] isn’t a factor. But, there are times I am just tired, or not feeling well from the medication that was supposed to cure my other ailment. I’m not a whiner, but it’s not always easy to hide my illness. I don’t want people to view me differently—more differently than I already view myself.’ [74] (p. 25). • Disclosure of mental or physical health problems was perceived as resulting in changed perceptions and potential disadvantage; ‘I feel a need to put on a grin and bounce around the department because I have seen things handed out to people on a silver platter and I want that too.’ [74] (p. 25). • DRs felt that poor responses to mental health disclosures might reflect universities trying to dissuade these DRs from continuing in academia; ‘You’ve noticed that I’ve been depressed for two years, and you’ve noticed that I couldn’t handle that other stuff, so why are you—are you purposely trying to just weed me out?’ [74] (p. 23).
	Emperor’s new clothes	• Supervisors often appeared to be the conduit for transmitting an academic ideal; ‘My advisor keeps telling me that [my family and I] should move for my job once I graduate because I will be the higher-earning spouse. But my husband is also successful. Yeah, I might make more money in my field, but my marriage has already suffered as a result of me pursuing my doctoral degree. I don’t think it can withstand a move that’s on me, for my career, regardless of how hard I’ve worked.’ [74] (p. 24). • DRs felt that valuing teaching was non-conformist and potentially dangerous; ‘While I do accept that research is important, I also believe teaching is equally so. But I can’t say it too much as it could jeopardize the chances of my contract being renewed.’ … One respondent mentioned the heightened anxieties that occurred due to a need to conform: Essentially, even when I don’t agree, I must comply or risk not finishing my program. As far as personal feelings, I live in a state of heightened anxieties, and need to conform to finish as quickly as humanly possible [55]. (p. 341). • DRs reported a dissonance between their personal and wider institutional values; ‘I struggle with the combination of my passion and ability. I can do the research, but do I enjoy the research to the level I need to if my entire professional career and reputation is based on it? I don’t know. I am struggling with that very question. But, God forbid I actually admit my thoughts to anyone.’ [74] (p. 21). • DRs reported feeling powerless and caught up in institutional values; ‘It’s easy to drink the Kool-Aid here, to buy into the mantra that I must aim to be a researcher in a top research university. At times, I find myself sucked into this. Other times, I feel resentful that the faculty in this department feel it’s their place to decide what we do with our lives.’ [74] (p. 21). • Feeling inauthentic when acting in line with institutional values had a high psychological toll; ‘The need to get along with a supervisor, to conform and to support institutional values was clearly expressed, and the use of such self-presentation behaviours created feelings of frustration, heightened anxieties and role stress—all which contribute to emotional exhaustion.’ [55] (p. 341). • DRs sensed disapproval when they acted in ways that could suggest values other than related to a research career; ‘I remember one telling me when I was pregnant that obviously I wasn’t taking it [her studies] seriously, and I thought, who the fuck is taking it more seriously? I mean, this is killing me.’ [76] (p. 93). • DRs felt unable to challenge institutional myths, such as the perceived institutional denial of the level of financial struggle involved in a PhD; ‘Students repeatedly explained that expenses and costs of living are not adequately covered by scholarships, stipends, tuition remission, and fellowships. One student asked, “So, how do they think people live?” These concerns led to some very expressive reactions to leaders’ suggestions concerning financial and budget-management counselling. It was deemed a “very elitist sentiment” by one, coming from a position of privilege, and that it was disenfranchising. The responses indicated that the problem is not knowing how to handle one’s finances but that the resources, no matter how well managed, are just not adequate to cover expenses.”’ [49] (p. 170).
	Beware the invisible and visible walls	• DRs felt that their success in academia rested on their ability to negotiate situational norms and rules; ‘…it was challenging to learn how to navigate the process, how to play the game.’ [39] (p. 87). • DRs felt vulnerable to being caught up in institutional conflicts; ‘…[if a student] is not aware of this minefield of political interests in the department, they can be hurt, not because people intend to, but just as a fallout of how faculty relate to each other’ [36] (p. 236). • DRs perceived academics and departments as poor at resolving conflicts; ‘…already during the postgraduate studies there are some things that could be thought of as ‘glass walls’ in the form of norms and values according to which the doctoral students learn to conduct themselves. This is demonstrated, for example, in how conflict resolution works in their own department. Most doctoral students did not think their own department handled conflicts in a good way. They mainly thought that conflicts were covered up and not properly aired. Sometimes they felt that there were unexpressed conflicts and unwritten rules with which they were unacquainted, and which had originated from old conflicts that were still present.’ [37] (p. 107-8). • DRs reported ambient anxiety and confusion over their own behaviour in the context of norm transgressions; ‘At seminars things happen that are difficult to comprehend until you have left the seminar. We had a horrible event, when this guy was completely humiliated by the opponent […] It got worse and worse. Nobody said anything. Nobody did anything. When we left the room it felt like we‘d participated in slaughtering him, and in a way we had, because nobody said anything.’ [37] (p. 105). • Gendered and racial micropolitics were evident; ‘So I think any student who wants to make a complaint or lodge anything formal, unless you're some white person who thinks or knows, hey, these institutions are here to serve me, then fine, let them go ahead. They are privileged to think that; I don't, because I know it'll be the opposite.’ [36] (p. 235). • Women and people of colour felt excluded or disadvantaged in visible and invisible ways; ‘It’s kind of elusive; it’s more a feeling somewhere in my head, so I can’t point to any specific events. It’s hard to know if it’s because I have a low position in the hierarchy or if it’s because I’m a woman […] but I often get this vague feeling that if I’d been a man, they wouldn’t interrupt me the way they do.’ [37] (p. 105-6). • It is possible to experience insider and outsider status simultaneously; ‘I am a[n African Canadian] man, these [faculty members] are European women’…she [the professor] and I went at it verbally in class… I didn't think it was worth the trouble [to make a formal complaint], and I thought that by virtue of being a man I was able to defend myself … She was stopped because I invoked male power’ [36] (p. 235). • Female DRs expressed feeling greater pressure and obligation both within and outside academia; ‘In the present study some of the informants reflected upon a kind of social responsibility. This is expressed, for example by Sanna: It’s especially the case for us female doctoral students. We can’t work if someone in the next room is crying. Some of the guys can’t either, while others don’t care about anything. They simply close their doors and write their thesis and now they’ve finished. That puts me under great stress’ [37] (p. 105). • Female DRs suggested they had to take on additional roles and responsibilities compared to males; ‘Different from males, we females are overburdened by house chores and other social life issues… When I try to compare myself, even though I have the capacity to do many things, I refrain from them as I have limited time to concentrate on my study. Even if I limited myself from other works which could have helped me get some more money, the time I have for my study is very much limited when compared to that of males’ [52] (p. 293). • Examples of successful women were those who had adopted a more traditional male role; ‘I think women in this field tend to be different from women at large. You know … I think they share a lot of the same traits as the males who are in this field. They are very driven’ [40] (p. 144).
4. Seeing, being and becoming		
	De-programming	• DRs spoke of rejecting the belief they should sacrifice personal relationships and identities; ‘One student in a male-dominated programme said she was not impressed by her advisor’s boasting of having only taken Christmas day off when he first began his career. She did not consider neglecting a family for a career as admirable. She and other students, mostly women, did not think they could devote themselves entirely to the tenure-track lifestyle. A number of men also stressed that it was important and often more enjoyable to be with their families than to be singularly focused on work.’ [40] (p. 150). • De-programming from prioritising academia above all was associated with greater confidence, career commitment and motivation; ‘…students reported the greatest confidence and motivation when faculty validated the importance of personal relationships and family matters, and helped students find a balance between their personal and professional lives.’ [40] (p. 152). • DRs suggested it possible to de-program from preoccupation with the ‘invisible walls’ of academia; ‘Certainly there is the [Professor A] camp and there is the [Professor B] camp and I am aware of that. … Has it affected me? No, I did my business [and] I try not to get involved in any conflicts. I don't know, some people would say I had my head in the sand, but, I mean, you have to kind of do what [you have to do]’ [36] (p. 236). • Interaction with people outside of academia was seen to scaffold de-programming; ‘It has been helpful to have this main girl [Caroline’s best friend] not be in my doctoral program so I can complain about it and she can give me an unbiased opinion’ [39] (p. 80). • De-programming also manifested as challenging perfectionistic beliefs; ‘I didn’t try to be the best; I tried to get through’ [40] (p. 146), and re-framing goals; ‘…you’ve gotta have your hands in several baskets and deal with them simultaneously and accept that all the baskets aren’t going to be nicely wrapped up in nice packages and on the due date’ [76] (p. 94). • Uncertainty can be re-framed as a privilege; ‘I have the right not to know yet…the privilege of asking questions and getting answers and supervision’ [81] (p. 41). • The PhD can be seen as an opportunity rather than a test; ‘Different doctoral students experience the academic field in different ways. For some, it feels like an arena with constantly ongoing battles, while for others it more closely resembles a pasture—a kind of a pasture of knowledge.’ [37] (p. 108). • Not completing the PhD can be re-framed as a viable life choice; ‘I know that I can go anytime that I want…Yeah it’s a coping mechanism [having “one foot out the door”]’ [76] (p. 81).
	The power of being seen	• DRs described powerful benefits to being seen: ▪ ‘An important component in the scientific room has to do with being seen—particularly by important key persons, “significant others”—people of importance to the informants on their journey towards a PhD degree. In the academic world this could be expressed as, “I’m seen, therefore I exist”’ [37] (p. 101). ▪ ‘Sanna focused mostly on the second aspect of the concept of seeing. In that her supervisor said that her area of research was important, she therefore felt some kind of security because she was seen by a key person at the department.’ [37] (p. 102). • Disciplinary communities could provide a needed sense of being seen; ‘Emily was the only participant, however, to speak about a strong sense of disciplinary community in Australia. By participating in conferences and co-authoring a journal article she obtained: E: … good feedback … it gives you this confidence and … I feel I'm being … taken care of in this [name of discipline] community very well in Australia. I don't know what happened where exactly it came but … I feel they … want to care about me. I don't feel it's everyone's case …I'm realising that … I'm in good hands and I have good people around me … they must—they believe in me, that's the thing.’ [42] (p. 180). • Positive engagement with the academic community was scaffolded by a sense of trust in the supervisor; ‘[I see the relation with the community] as both responsible and challenging but on the other hand as safe, because I fully trust my supervisor’s ability to evaluate the quality of my work.’ [81] (p. 41). • Spending time with peers could provide a sense of shared experience; ‘Betty continued, “We have been afforded this beautiful opportunity to walk beside each other through this program and that is something I don’t take for granted and neither does she.”’ [39] (p. 79). • Friendship was seen to buffer stress and protect against mental health problems; ‘…an intimate, best friendship may function as a mental health protective factor due to the best friend’s capacity to recognize any emerging mental health issues of their friend who is immersed in doctoral study, even if they, too, are enrolled in a doctoral program, based upon the familiarity and camaraderie within the relationship. Such protective elements of having a best friendship were detailed by the participants through the following statements: [Allen]: “My best friend knows me very well, he knows my history very well”. [Betty]: “She is very consistent and faithful, she is very intentional and following up with things, she always asks me how I’m doing. She knows how to dig deeper and to get me to really open up and share my feelings.”’ [39] (p. 94). • Designated physical spaces seem important to being seen; ‘In her story, Kim described a feeling of being deserted when she began her research. She said it took at least two years before she grasped what she was supposed to do, and that this had felt horrible. Gradually, however, the situation changed. Kim started to spend more time at the department and established a physical presence there—she took over an empty desk—and she began to be seen more. She established more contact with others at the department and started to meet her supervisor more often.’ [37] (p. 102). • Peers within the university could provide physical embodiments of being seen: ▪ ‘Marianne, who initially had not belonged to a research group but who was now a part of a group, described different examples indicating that she now felt a sense of belonging. One example was when she had been on holiday, and found a sign saying ‘Welcome home’ when she came back.’ [37] (p. 104). ▪ ‘[Interviewer: I see a photo of cardboard boxes] Those aren’t cardboard boxes - that is a castle! Can’t you see the castle!? (laughs) It has the arms symbol over it. While I was away on vacation it was my birthday, and when I came back the next week they [lab mates] had built this castle for me for a birthday present..’ [50] (p. 204). • Close others outside the university can support DRs’ authenticity; ‘She is someone I can be myself with and not being concerned with formalities or expectations.’ [50] (p. 205). • Pets could support feeling seen without requiring the expenditure of too much energy; ‘Pets provided love and support to students, without charging them for their time and without excessive communication… Pets made students feel special and important, at times when they might have felt insignificant and unappreciated..’ [50] (p. 207). • With sufficient self-agency, DRs can see themselves and render themselves seen; ‘Marianne had held a seminar concerning her research. She described her experience of holding a seminar as rather affirmative. At the seminar Marianne was seen; people showed interest in what she said, and relevant questions were posed.’ [37] (p. 104).
	Multiple goals, roles and groups	• Multiple role and activities appeared essential for protecting against mental health problems; ‘We could all very well do nothing but our school work and worry about presentations and like conferences, but we would probably go crazy and you would never get out of this program because the stress and everything else would probably kill you.’ [68] (p. 50). • Leisure activities appear to support mental health through promoting physical health, buffering stress, uplifting mood and offering multiple identities; ‘…one (112, Latino male) reflected, “A lot of DRs forget to take care of themselves; I see it a lot. Science is important, but your body is more important.” …Activities served as an emotional and mental release for many doctoral learners, using informal and formal activities, such as fitness classes, dance, and television as their forms of catharsis. … Representative comment included: I am actually a part of the dance company, a ballet company on campus, …. so I might be a scientist, but I’m also a dancer, and I need to have time to be able to do that (Participant 117, Asian/Latina female, describing a photo of dancers’ legs).’ [50] (p. 207). • Competing roles could support psychological separation from the PhD by requiring physical separation: ▪ ‘I go home and I leave work…I have a family’ [59] (p. 10). ▪ ‘…pets gave students someone to come home to, and forced them to venture outside of the laboratory and embrace nature and obligations beyond their PhD program.’ [50] (p. 207). • Enjoyable and self-care activities provided a sense of balance and normalcy: ▪ ‘I just can’t emphasize enough the importance of balance. That is probably the biggest lesson I’ve learned so far. Through balance you maintain good, positive mental health.’ [39] (p. 80). ▪ ‘…a work-life balance has to exist for your health and your sanity.’ [68] (p. 48). • All DRs appeared to benefit from treating the PhD as only one aspect of life; ‘On the other hand, for Stephanie, who is “not currently romantically involved,” well-being was maintained when she participated in activities “as if I have a life outside of the work I’m doing.”’ [59] (p. 10). • Additional roles and activities render not completing the PhD as less averse; ‘A woman in a gender-balanced programme with one child and a second expected said she was less stressed than others in her programme without families because she was ‘not putting all my eggs into one basket’.’ [40] (p. 148).
	Finding hope, meaning and authenticity	• Finding hopefulness and meaning in the PhD can scaffold purpose, enjoyment, and authenticity; ‘The background is chaotic, right? Chaotic, a lot of motion, and then in the foreground is a caravan moving through the desert and everything seems serene and ordered, and you see the stars and so much beauty. And I think this is how I would describe myself, that I am on a pilgrimage, like the caravan in the desert, and I strive to be at peace and have a sense of order in the midst of a world that is very chaotic and seems to be moving around seemingly without purpose. And I think I strive to be an example of the fact that we do have a sense of purpose and we are moving toward it very slowly but with purposefully and with great peace and serenity. (Participant 190, Asian male)’ [50] (p. 210). • Hopefulness is predicated on identifying a goal; ‘Stephanie states, “I can see myself walking across the stage” which gives Stephanie a larger view of her actions in a positive light that affects her well-being in a positive manner.’ [59] (p. 11). • Hopefulness is enhanced by breaking tasks down into small steps; ‘…if I bit off little chunks of the elephant, as my mum says, bit by bit, it won’t seem as big an obstacle’ [38] (p. 128). • Meaning manifested as passion in action; ‘But nothing’s as good as the high of ideas’ [76] (p. 103). • Meaning also manifested as DRs feeling that they were living in accordance with their values and had a sense of purpose; ‘Makaila stated, “There are bigger things out there.” This would suggest that she under-stands her purpose is a larger than just her doctoral studies and would encompass more. Makaila also stated “attend to the family” as a way to balance her well-being, but it also displays her placing importance of having a larger life than just her graduate school aspirations.’ [59] (p. 11). • Feeing that supervisors believed in them scaffolded DRs sense of self-agency and motivation; ‘Journey explained that his supervisors’ trust in his ability had given him the confidence to begin writing: J: when … you are a person from an environment that is not really value publication as it is here, and then you come to this place – R: – to compete on an even footing – J: Yes … I was very happy that my supervisors yeah they trust me, tried to motivate me – ‘Yes you can, you have experience’ and … when we discuss content-based knowledge, ah maybe they said – ‘Yeah … you have’ R: Mmm J: Then ah one of them at that time started to ask me to write a paper. I guess it's a kind of recognition that you … can do that. So, yeah that's part of things that strengthened myself that I … could do.’ [42] (p. 181). • Other people could motivate DRs to finish; ‘Jenny: “Even when I wanted to quit, even though she was sympathetic and understanding, she quickly told me, “No, not on your life.”’ Katie: ‘To be perfectly honest, if it were not for my best friend, I probably would have either dropped out or failed out of my program. I could only get through this with him and that is essential.’ [39] (p. 92). • Meaning appeared scaffolded by contribution, belonging and mattering: ▪ ‘Through his PhD, Journey hopes to convince his Indonesian colleagues that they too can participate in international research: I should communicate … with them … remind them that it's all about efforts, it's all about ah commitment … maybe come up with failures, but you have to try. … So I've done my part, though it's small and shows us that yes we can! (Journey, 6, 1036–1044)’ [42] (p. 184). ▪ ‘I use encouragement as a way to invest in other people and I feel like I’ve received that too. Words and encouragement like, “You’re not alone in this,” are very meaningful and helpful.’ [39] (p. 79). • DRs could use agentic action to source a community for collective authenticity; ‘…if you want to be cutthroat and hateful to people then you can be, but if you want to have an engaging, enriching educational experience, there are people out there who want that same thing.’ [39] (p. 88).
	The PhD as a process of transcendence	• The PhD acted as a forge, testing and remoulding DRs into something greater; ‘…[my supervisor’s] office is a little bit intimidating to go in there, but it is also a very challenging and intellectually stimulating place, and it is where a lot of really good conversations happen, and it feels like you are really pushing your limits every time you are in there having conversations with him. (Participant 24, American Indian/White female)’ [50] (p. 202-3). • The struggle caused DRs to have a greater sense of their capacities; ‘…this semester it was like everything else was thrown at me, and I feel like I have stood tall, and nothing at this point can really deter me from my goal of getting my PhD (Participant 64, Black male)’ [50] (p. 210). • A trusted supervisor aids in the process of transcendence; ‘He has really been there for me to go through the growing pains of learning how to think and learn differently.’ [62] (p. 49). • The PhD could allow DRs to transcend personal tragedy; ‘Regarding mental health, from the outset of my program, academics became a productive outlet through which I channelled personal stress and frustration; … I recently described my doctoral experience as “merely a former coping mechanism that has evolved into an oasis of creativity and scholarly enrichment.”’ [39] (p. 88). • The PhD could serve as a transformative selection process for social relationships; ‘It is good to test a relationship,’ he asserted, ‘Challenges, I believe, validate our relationship.’ [39] (p. 84), with some relationships cast aside, ‘It is good to test a relationship,’ he asserted, ‘Challenges, I believe, validate our relationship.’ [39] (p. 84), and some forged anew; ‘…many of my best friends went through the program with me and I feel much closer to these friends than my other friends who were not in the program because of the bonding experiences we have shared.’ [39] (p. 85).

##### Always alone in the struggle

‘Always alone in the struggle’ reflects the isolated nature of the PhD experience. Two subthemes reflect different aspects of being alone; ‘Invisible, isolated and abandoned’ represents DRs’ sense of physical and psychological separation from others and ‘It’s not you, it’s me’ represents DRs’ sense of being solely responsible for their PhD process and experience.

##### Invisible, isolated and abandoned

Feeling invisible and isolated both within and outside of the academic environment appears a core DR experience [39, 43, 81]. Isolation from academic peers seemed especially salient for DRs with less of a physical presence on campus, e.g. part-time and distance students, those engaging in extensive fieldwork, outside employment and those with no peer research or lab group [36, 52, 68]. Where DRs reported relationships with DR peers, these were characterised as low quality or ‘not proper friendships’ and this appeared linked to a sense of essential and obvious competition amongst DRs with respect to current and future resources, support and opportunities [39], in which a minority of individuals were seen to receive the majority share [36, 74]. Intimate sharing with peers thus appeared to feel unsafe. This reflected the competitive environment but also a sense of peer relationships being predicated on too shared an experience [39].

In addition to poor peer relations, a mismatch between the expected and observed depth of supervisor interest, engagement and was evident [40, 81]. This mismatch was clearly associated with disappointment and anger, and a sense of abandonment, which appeared to impact negatively on DR mental health and wellbeing [42] (p. 182). Moreover, DRs perceived academic departments as complicit in their isolation; failing to offer adequate opportunities for academic and social belonging and connections [42, 81] and including PGRs only in a fleeting or ‘hollow’ sense [37]. DRs identified this isolation as sending a broader message about academia as a solitary and unsupported pursuit; a message that could lead some DRs to self-select out of planning for future in academia [37, 42]. DRs appeared to make sense of their lack of belonging in their department as related to their sense of being different, and that this difference might suggest they did not ‘fit in’ with academia more broadly [74]. In the short-term, DRs might expend more effort to try and achieve a social and/or professional connection and equitable access to support, opportunities and resources [74]. However, over the longer-term, the continuing perception of being professionally ‘other’ also seemed to undermine DRs’ sense of meaning and purpose [81] and could lead to opting out of an academic career [62, 74].

Isolation within the PhD was compounded by isolation from one’s personal relationships. This personal isolation was first physical, in which the laborious nature of the PhD acted as a catalyst for the breakdown of pre-existing relationships [76]. Moreover, DRs also experienced a sense of psychological detachment [45, 74]. Thus, the experience of isolation appeared to be extremely pervasive, with DRs feeling excluded and isolated physically and psychologically and across both their professional and personal lives.

##### It’s not you, it’s me

‘It’s not you, it’s me’ reflects DRs’ perfectionism as a central challenge of their PhD experience and a contributor to their sense of psychological isolation from other people. DRs’ perfectionism manifested in four key ways; firstly, in the overwhelming sense of responsibility experienced by DRs; secondly, in the tendency to position themselves as inadequate and inferior; thirdly, in cycles of perfectionist paralysis; and finally, in the tendency to find evidence which confirms their assumed inferiority.

DRs positioned themselves as solely responsible for their PhD and for the creation of a positive relationship with their supervisor [36, 52, 81]. DRs expressed a perceived need to capture their supervisors’ interest and attention [36, 52, 74], feeling that they needed to identify and sell to their supervisors some shared characteristic or interest in order to scaffold a meaningful relationship. DRs appeared to feel it necessary to assume sole responsibility for their personal lives and to prohibit any intrusion of the personal in to the professional, even in incredibly distressing circumstances [42].

DRs appeared to compare themselves against an ideal or archetypal DR and this comparison was typically unfavourable [37], with DRs contrasting the expected ideal self with their actual imperfect and fallible self [37, 42, 52]. DRs’ sense of inadequacy appeared acutely and frequently reflected back to them by supervisors in the form of negative or seemingly disdainful feedback and interactions [41, 76]. DRs framed negative supervisor responses as a cue to work harder, meaning they were continually striving, but never reaching, the DR ideal [76]. This ideal-actual self-discrepancy was associated with a tendency towards punitive self-talk with clear negative valence [38].

DRs appear to commonly use self-castigation as a necessary (albeit insufficient) means to motivate themselves to improve their performance in line with perfectionistic standards [38, 41]. The oscillation between expectation and actuality ultimately resulted in increased stress and anxiety and reduced enjoyment and motivation. Low motivation and enjoyment appeared to cause procrastination and avoidance, which lead to a greater discrepancy between the ideal and actual self; in turn, this caused more stress and anxiety and further reduced enjoyment and motivation leading to a sense of stuckness [76].

The internalisation of perceived failure was such that DRs appeared to make sense of their place, progress and possible futures through a lens of inferiority, for example, positioning themselves as less talented and successful compared to their peers [37]. Thus, instances such as not being offered a job, not receiving funding, not feeling connected to supervisors, feeling excluded by academics and peers were all made sense of in relation to DRs’ perceived relative inadequacy [36].

##### Death of personhood

The higher-order theme ‘Death of personhood’ reflects DRs’ identity conflict during the PhD process; a sense that DRs’ engage in a ‘Sacrifice of personal identity’ in which they feel they must give up their pre-existing self-identity, begin to conceive of themselves as purely ‘takers’ personally and professionally, thus experiencing the ‘Self as parasitic’, and ultimately experience a ‘Death of self-agency’ in relation to the thesis, the supervisor and other life roles and activities.

##### A sacrifice of personal identity

The sacrifice of personal identity first manifests as an enmeshment with the PhD and consequent diminishment of other roles, relationships and activities that once were integral to the DRs’ sense of self [59, 76]. DRs tended to prioritise PhD activities to the extent that they engaged in behaviours that were potentially damaging to their personal relationships [76]. DRs reported a sense of never being truly free; almost physically burdened by the weight of their PhD and carrying with them a constant ambient guilt [37, 38, 44, 76]. Time spent on non-PhD activities was positioned as selfish or indulgent, even very basic activities of living [76].

The seeming incompatibility of aspects of prior personal identity and the PhD appears to result in a sense of internal conflict or identity ‘collision’ [59]. Friends and relatives often provided an uncomfortable reflection of the DR’s changing identity, leaving DRs feeling hyper-visible and carrying the burden of intellect or trailblazer status [74]; providing further evidence for the incompatibility of their personal and current and future professional identities. Some DRs more purposefully pruned their relationships and social activities; to avoid identity dissonance, to conserve precious time and energy for their PhD work, or as an acceptance of total enmeshment with academic work as necessary (although not necessarily sufficient) for successful continuation in academia [40, 52, 77]. Nevertheless, the diminishment of the personal identity did not appear balanced by the development of a positive professional identity. The professional DR identity was perceived as unclear and confusing, and the adoption of an academic identity appeared to require DRs to have a greater degree of self-assurance or self-belief than was often the case [37, 81].

##### Self as parasitic

Another change in identity manifested as DRs beginning to conceive of themselves as parasitic. DRs spoke of becoming ‘takers’, feeling that they were unable to provide or give anything to anyone. For some DRs, being ‘parasitic’ reflected them being on the bottom rung of the professional ladder or the ‘bottom of the pile’; thus, professionally only able to receive support and assistance rather than to provide for others. Other DRs reported more purposefully withdrawing from activities in which they were a ‘giver’, for example voluntary work, as providing or caring for others required time or energy that they no longer had [38, 44]. Furthermore, DRs appeared to conceive of themselves as also causing difficulty or harm to others [81], as problems in relation to their PhD could lead them to unwillingly punishing close others, for example, through reducing the duration or quality of time spent together [38].

Feeling that close others were offering support appeared to heighten the awareness of the toll of the PhD on the individual and their close relationships, emphasising the huge undertaking and the often seemingly slow progress, and actually contributing to the sense of ambient guilt, shame, anger and failure [38]. Moreover, DRs spoke of feeling extreme guilt in perceiving that they had possibly sacrificed their own, and possibly family members’, current wellbeing and future financial security [49].

##### Death of self-agency

In addition to their sense of having to sacrifice their personal identity, DRs also expressed a loss of their sense of themselves as agentic beings. DRs expressed feeling powerless in various domains of their lives. First, DRs positioned the thesis as a powerful force able to overwhelm or swallow them [46, 52, 59]. Secondly, DRs expressed a sense of futility in trying to retain any sense of personal power in the climate of academia. An acute feeling of powerlessness especially in relation to supervisors was evident, with many examples provided of being treated as means to an end, as opposed to ends in themselves [39, 42, 62]. Supervisors did not interact with DRs in a holistic way that recognised their personhood and instead were perceived as prioritising their own will, or the will of other academics, above that of the DR [39, 62].

Furthermore, DRs reported feeling as if they were used as a means for research production or furthering their supervisors’ reputations or careers [62]. DRs perceived that holding on to a sense of personal agency sometimes felt incompatible with having a positive supervisor relationship [42]. Thus whilst emotional distress, anger, disappointment, sadness, jealousy and resentment were clearly evident in relation to feeling excluded, used or over-powered by supervisors [37, 42, 52, 62], DRs usually felt unable to change supervisor irrespective of how seriously this relationship had degraded [37, 62]. Instead, DRs appeared to take on a position of resignation or defensive pessimism, in which they perceived their supervisors as thwarting their personhood, personal goals and preferences, but typically felt compelled to accept this as the status quo and focus on finishing their PhDs [42]. DRs resignation was such that they internalised this culture of silence and silenced themselves; tending to share litanies of problems with supervisors whilst prefacing or ending the statements with some contradictory or undermining phrase such as ‘but that’s okay’ [42, 52].

The apparent lack of self-agency extended outward from the PhD into DRs not feeling able to curate positive life circumstances more generally [76]. A lack of time was perhaps the key struggle across both personal and professional domains, yet DRs paradoxically reported spending a lot of time procrastinating and rarely (if ever) mentioned time management as a necessary or desired coping strategy for the problem of having too little time [46]. The lack of self-agency was not only current but also felt in reference to a bleak and uncertain future; DRs lack of surety in a future in academia and the resultant sense of futility further undermined their motivation to engage currently with PhD tasks [38, 40].

##### The system is sick

The higher-order theme ‘The system is sick’ represents systemic influences on DR mental health. First, ‘Most everyone’s mad here’ reflects the perceived ubiquity mental health problems amongst DRs. ‘Emperor’s new clothes’ reflects the DR experience of engaging in a performative piece in which they attempt to live in accordance with systemic rather than personal values. Finally, ‘Beware the invisible and visible walls’ reflects concerns with being caught between ephemeral but very real institutional divides.

##### Most everyone’s mad here

No studies focused explicitly on experiences of DRs who had been given diagnoses of mental health problems. Some study participants self-disclosed mental health problems and emphasised their pervasive impact [50]. Further lived experiences of mental distress in the absence of explicit disclosure were also clearly identifiable. The ‘typical’ presentation of DRs with respect to mental health appeared characterised as almost unanimous [39] accounts of chronic stress, anxiety and depression, emotional distress including frustration, anger and irritability, lack of mental and physical energy, somatic problems including appetite problems, headaches, physical pain, nausea and problems with drug and alcohol abuse [39, 46, 59, 76]. Health anxiety, concerns regarding perceived new and unusual bodily sensations and perceived risks of developing stress-related illnesses were also common [46, 59, 76]. A PhD-specific numbness and hypervigilance was also reported, in which DRs might be less responsive to personal life stressors but develop an extreme sensitivity and reactivity to PhD-relevant stimuli [39].

An interplay of trait and state factors were suggested to underlie the perceived ubiquity of mental health problems amongst DRs. Etiological factors associated with undertaking a PhD specifically included the high workload, high academic standards, competing personal and professional demands, social isolation, poor resources in the university, poor living conditions and poverty, future and career uncertainty [36, 41, 43, 46, 49, 76]. The ‘nexus’ of these factors was such that the PhD itself acted as a crucible; a process of such intensity that developing mental health problems was perhaps inevitable [39].

The perceived inevitability of mental health problems was such that DRs described people who did not experience mental health problems during a PhD as ‘lucky’ [39]. Supervisors and the wider academic system were seen to promote an expectation of suffering, for example, with academics reportedly normalising drug and alcohol problems and encouraging unhealthy working practices [39]. Furthermore, DRs felt that academics were uncaring with respect to the mental challenge of doing a PhD [39]. Nevertheless, academics were suggested to deny any culpability or accountability for mental health problems amongst DRs [39, 59, 74]. The cycle of indigenousness was further maintained by a lack of mental health literacy and issues with awareness, availability and access to help-seeking and treatment options amongst DRs and academics more widely [39]. Thus, DRs appeared to feel they were being let down by a system that was almost set up to cause mental distress, but within which there was a widespread denial of the size and scope of the problem and little effort put into identifying and providing solutions [39, 59]. DRs ultimately felt that the systemic encouragement of unhealthy lifestyles in pursuit of academic success was tantamount to abuse [62].

##### A performance of optimum suffering

Against a backdrop of expected mental distress, DRs expressed their PhD as a performative piece. DRs first had to show just the right amount of struggle and difficulty; feeling that if they did not exhibit enough stress, distress and ill-health, their supervisors or the wider department might not believe they were taking their PhD seriously enough [40]. At the same time, DRs felt that their ‘researcher mettle’ was constantly being tested and they must rise to this challenge. This included first guarding against presenting oneself as intellectually inferior [36]. Yet it also seemed imperative not to show vulnerability more broadly [74]. Disclosing mental or physical health problems might lead not only to changed perceptions of the DR but to material disadvantage [74]. The poor response to mental health disclosures suggested to some DRs that universities might be purposefully trying to dissuade or discourage DRs with mental health problems or learning disabilities from continuing [74]. The performative piece is thus multi-layered, in that DRs must experience extreme internal psychological struggles, exhibit some lower-level signs of stress and fatigue for peer and faculty observance, yet avoid expressing any real academic or interpersonal weakness or the disclosure of any diagnosable disability or disease.

##### Emperor’s new clothes

DRs described feeling beholden to the prevailing culture in which it was expected to prioritise above all else developing into a competitive, self-promoting researcher in a high-performing research-active institution [39, 42]. Supervisors often appeared the conduit for transmission of this academic ideal [74]. DRs felt reticent to act in any way which suggested that they did not personally value the pursuit of a leading research career above all else. For example, DRs felt that valuing teaching was non-conformist and could endanger their continuing success within their current institution [55]. Many DRs thus exhibited a sense of dissonance as their personal values often did not align with the institutional values they identified [74]. Yet DRs expressed a sense of powerlessness and a feeling of being ‘caught up’ in the values of the institution even when such values were personally incongruent [74]. The psychological toll of this sense of inauthenticity seemed high [55]. Where DRs acted in ways which ostensibly suggested values other than prioritising a research career, for example becoming pregnant, they sensed disapproval [76]. DRs also felt unable to challenge other ‘institutional myths’ for example, the perceived institutional denial of the duration of and financial struggle involved in completing a PhD [49]. There was a perceived tendency of academics to locate problems within DRs as opposed to acknowledging institutional or systemic inequalities [49]. DRs expressed strongly a sense in which there is inequity in support, resources and opportunities, yet universities were perceived as ignoring such inequity or labelling such divisions as based on meritocracy [36, 74].

##### Beware the invisible and visible walls

DRs described the reality of working in academia as needing to negotiate a maze of invisible and visible walls. In the former case, ‘invisible walls’ reflect ephemeral norms and rules that govern academia. DRs felt that a big part of their continuing success rested upon being able to negotiate such rules [39]. Where rules were violated and explicit or implicit conflicts occurred, DRs were seen to be vulnerable to being caught in the ‘crossfire’ [36]. DRs identified academic groups and departments as being poor in explicitly identifying, discussing and resolving conflicts [37]. The intangibility of the ‘invisible walls’ gave rise to a sense of ambient anxiety about inadvertently transgressing norms and divides, such that some DRs reported behaving in ways that surprised even themselves [37].

Gendered and racial micropolitics of academic institutions were seen to manifest as more visible walls between people, with institutions privileging those with ‘insider’ status [36]. Women and people of colour typically felt excluded or disadvantaged in a myriad of observable and unobservable ways, with individuals able to experience both insider and outsider statuses simultaneously [36, 37], for example when a male person of colour [36]. Female DRs suggested that not only must women prove themselves to a greater extent than men to receive equal access to resources, opportunities and acclaim but also are typically under additional pressure in both their professional and personal lives [37, 52, 76]. Women also felt that they had to take on more additional roles and responsibilities and encountered more conflicts in their personal lives compared to men [52]. Examples of professionally successful women in DRs’ departments were described as those who had crossed the divide and adopted a more traditionally male role [40]. Thus, being female or non-White were considered visible characteristics that would disadvantage people in the competitive academic environment and could give rise to a feeling of increased stress, pressure, role conflicts, and a feeling of being unsafe.

##### Seeing, being and becoming

The higher-order theme of ‘Seeing, being and becoming’ reflects protective and transformative influences on DR mental health. ‘De-programming’ refers to the DRs disentangling their personal beliefs and values from systemic values and also from their own tendency towards perfectionism. ‘The power of being seen’ reflects the positive impact on DR mental health afforded by feeling visible to personal and professional others. ‘Finding hope, meaning and authenticity’ refers to processes by which DRs can find or re-locate their own self-agency, purpose and re/establish a sense of living in accordance with their values. ‘The importance of multiple goals, roles and groups’ represents the beneficial aspects of accruing and sustaining multiple aspects to one’s identity and connections with others and activities outside the PhD. Finally, ‘The PhD as a process of transcendence’ reflects how the struggles involved in completing a PhD can be transformative and self-actualising.

##### De-programming

DRs reported being able to protect their mental health by ‘de-programming’ and disentangling their attitudes and practices from social and systemic values and norms. This disentangling helped negate DRs’ adopting unhealthy working practices and offered some protection against experiencing inauthenticity and dissonance between personal and systemic values.

First, DRs spoke of rejecting the belief that they should sacrifice or neglect personal relationships, outside interests and their self-identity in pursuit of academic achievement. DRs could opt-out entirely by choosing a ‘user-friendly’ programme [44] which encouraged balance between personal and professional goals, or else could psychologically reject the prevailing institutional discourse [40]. Rather than halting success, de-programming from the prioritisation of academia above all else was seen to be associated not only with reduced stress but greater confidence, career commitment and motivation [40, 50]. It was also suggested possible to ‘de-programme’ in the sense of choosing not to be preoccupied by the ‘invisible walls’ of academia and psychologically ‘opt out’ of being concerned by potential conflicts, norms and rules governing academic workplace conduct [36]. Interaction with people outside of academia was seen to scaffold de-programming, by helping DRs to stay ‘grounded’ and offering a model what ‘normal’ life looks like. People outside of academia could also help DRs to see the truth by providing unbiased opinions regarding systemic practices [39].

A further way in which de-programming manifested was in DRs challenging their perfectionist beliefs. This include re-framing the goal as not trying to be the archetype of a perfect DR, and accepting that multiple demands placed on one individual invariably requires compromise [40, 76]. DRs spoke of the need to conceptualise the PhD as a process, rather than just a product [46, 82]. The process orientation facilitated framing of the PhD as just one-step in the broader process of becoming an academic as opposed to providing discrete evidence of worth [82]. Within this perspective, uncertainty itself could be conceived as a privilege [81]. The PhD was then seen as an opportunity rather than a test [37, 46]. Moreover, the process orientation facilitated viewing the PhD as a means of growing into a contributing member of the research community, as opposed to needing to prove oneself to be accepted [82]. Remembering the temporary nature of the PhD was advised [45] as was holding on to a sense that not completing the PhD was also a viable life choice [76]. DRs also expressed, implicitly or explicitly, a decision to change their conceptualisation of themselves and their progress; choosing not to perceive themselves as stuck, but planning, learning and progressing [38, 39, 81, 82]. This new perspective appeared to be helpful in reducing mental distress.

##### The power of being seen

DRs described powerful benefits to feeling seen by other people, including a sense of belonging and mattering, increased self-confidence and a sense of positive progress [37]. Being seen by others seems to provoke the genesis of an academic identity; it brings DRs into existence as academics. Being seen within the academic institution also supports mental health and can buffer emotional exhaustion [37, 52, 55, 81]. DRs expressed a need to feel that supervisors, academics and peers were interested in them as people, their values, goals, struggles and successes; yet they also needed to feel that they and their research mattered and made a difference within and outside of the institution [42, 52, 81]. It was clear that DRs could find in their disciplinary communities the sense of belonging that often eluded them within their immediate departments [42]. Feeling a sense of belonging to the academic community seemed to buffer disengagement and amotivation during the PhD [81]. Positive engagement with the broader community was scaffolded by a sense of trust in the supervisor [81]. DRs often felt seen and supported by postdocs, especially where supervisors appeared absent or unsupportive [50].

Spending time with peers could be beneficial when there was a sense of shared experience and walking alongside each other [39]. Friendship was seen to buffer stress and protect against mental health problems through the provision of social and emotional support and help in identifying struggles [39, 43]. In addition to relational aspects, the provision of designated physical spaces on campus or in university buildings also seemed important to being seen [37]. Peers in the university could provide DRs with further physical embodiments of being seen, for example, gift-giving in response to their birthdays or returning from leave [37, 50]. Outside of the academic institution, DRs described how being seen by close others could support DRs to be their authentic selves, providing an antidote to the invisible walls of academia [50]. Good quality friendships within or outside academia could be life-changing, providing a visceral sense of connection, belonging and authenticity that can scaffold positive mental health outcomes during the PhD [39]. Pets could also serve to help DRs feel seen but without needing to extend too much energy into maintaining social relationships [50].

Finally, DRs also needed to see themselves, i.e. to begin to see themselves as burgeoning academics as opposed to ‘just students’ [81]. Feeling that the supervisor and broader academic community were supportive, developing one’s own network of process collaborators and successfully obtaining grant funding seemed tangible markers that helped DRs to see themselves as academics [37, 81]. Seeing their own work published was also helpful in providing a boost in confidence and being a joyful experience [42]. Moreover, with sufficient self-agency, DRs can not only see themselves but render themselves visible to other people [37].

##### Multiple goals, roles and groups

In antidote to the diminished personal identity and enmeshment with the PhD, DRs benefitted from accruing and sustaining multiple goals, roles, occupations, activities and social group memberships. Although ‘costly’ in terms of increased stress and role conflicts, sustaining multiple roles and activities appeared essential for protecting against mental health problems [50, 68].

Leisure activities appeared to support mental health through promoting physical health, buffering stress, providing an uplift to DRs’ mood and through the provision of another identity other than as an academic [44, 50, 76]. Furthermore, engagement in activities helped DRs to find a sense of freedom, allowing them to carve up leisure and work time and psychologically detach from their PhD [68, 76]. Competing roles, especially family, forced DRs to distance themselves from the PhD physically which reinforced psychological separation [50, 59]. Engaging in self-care and enjoyable activities provided a sense of balance and normalcy [39, 44, 68]. This normalcy was a needed antidote to abnormal pressure [59]. Even in the absence of fiercely competing roles and priorities, DRs still appeared to benefit from treating their PhD as if it is only one aspect of life [59]. Additional roles and activities reduced enmeshment with the PhD to the extent that considering not completing the PhD was less averse [40]. This position appeared to help DRs to be less overwhelmed and less sensitive to perceived and anticipated failures.

##### Finding hope, meaning and authenticity

Finding hopefulness and meaning within the PhD can scaffold a sense of living a purposeful, enjoyable, important and authentic life. Hopefulness is predicated on the ability to identify a goal, i.e. to visualise and focus on the desired outcome and to experience both self-agency and potential pathways towards the goal. Hopefulness was enhanced by the ability to break down tasks into smaller goals and progress in to ‘baby steps’ [38, 59]. In addition, DRs benefitted from finding explicit milestones against which they can compare their progress [59], as this appeared to feed back into the cycle of hopeful thinking and spur further self-agency and goal pursuit.

The experience of meaning manifested in two main ways; first as the more immediate lived experience of passion in action [76]. Secondly, DRs found meaning in feeling that in their PhD and lives more broadly they were living in accordance with their values, for example, experiencing their own commitment in action through continuing to work on their PhD even when it was difficult to do so [76]. DRs who were able to locate their PhD within a broader sense of purpose appeared to derive wellbeing benefits. There was a need to ensure that values were in alignment, for example, finding homeostasis between emotional, intellectual, social and spiritual parts of the self [46, 59, 90].

The processes of finding hopefulness and meaning appear to be largely relational. Frequent contact with supervisors in person and social and academic contact with other DRs were basic scaffolds for hope and meaning [52]. DRs spoke of how a sense that their supervisors believed in them inspired their self-agency and motivation [42, 62, 76]. Partners, friends and family could also inspire motivation for continuing in PhD tasks [44, 76]. Other people also could help instil a sense of motivation to progress and complete the PhD; a sense of being seen is to be beholden to finish [39]. Meaning appeared to be scaffolded by a sense of contribution, belonging and mattering [81] and could arise from the perception of putting something into the collective pot, inspiring hopefulness and helping others [39, 42]. Moreover, hopefulness, meaning and authenticity also appeared mutually reinforcing [81]. Finding meaning and working on a project which is in accordance with personal values, preferences and interests is also helpful in completing the PhD and provides a feedback loop into hope, motivation and agentic thinking [39, 81]. Furthermore, DRs could use agentic action to source a community of people who share their values, enabling them to engage in collective authenticity [39].

##### The PhD as a process of transcendence

The immense challenge of the PhD could be a catalyst for growth, change and self-actualisation, involving empowerment through knowledge, self-discovery, and developing increased confidence, maturity, capacity for self-direction and use of one’s own autonomy [44, 82]. The PhD acted as a forge in which DRs were tested and became remoulded into something greater than they had been before [44, 82, 90]. The struggles endured during the PhD caused DRs to reconsider their sense of their own capacities, believing themselves to be more able than they previously would have thought [50]. The struggles endured added to the sense of accomplishment. A trusted and trusting supervisor appears to aid in the PhD being a process of transcendence [62].

More broadly, the PhD also helped DRs to transcend personal tragedy, allowing immersion in a meaningful activity which begins as a means of coping and becomes something completely [39]. The PhD could also serve as a transformative selection process for DRs’ social relationships, with some relationships cast aside and yet others formed anew [39]. Overall, therefore, the very aspects of the PhD which were challenging, and distressing could allow DRs to transcend their former selves and, through the struggle, become something more.

### Summation of results

The findings regarding the risk and protective factors associated with DR mental health have been summarised in Table 3 in relation to (1) the type of research evidencing the factor (i.e. whether the evidence is quantitative only, part of the meta-synthesis only, or evident in both results sections); and (2) the volume of evidence (i.e. whether the factor was found in a single study or across multiple studies). The factors in the far-right column (i.e. the factors found across multiple research studies utilising both qualitative and quantitative methods) are the ones with the strongest evidence at present.

**Table 3:** Risk and protective factors associated with DR mental health in terms of the type and volume of evidence

	Quantitative only		Qualitative only		Both
	Single	Multiple	Single	Multiple	
Risk	Being single. Not having children Lower socio-economic status In final year Viewed studies as a burden Study-related self-efficacy	Uncertainty. Negative writing habits Difficulties in personal life	Competitiveness amongst peers. Lack of academic identity Pre-existing mental health problems Dissonance between personal and institutional values	No physical presence on campus Poor quality friendships Feeling like an outsider Imposter phenomenon Perfectionism Putting studies above all else Feeling like a product Powerlessness Non-white Unhealthy work culture	Being female Isolation
Protective	Positive writing habits	Five factor personality traits. Self-concept	Support from postdoctoral researchers	Involvement in non-PhD activities Being part of multiple groups (inside and outside of academia) Being authentic Seeing the bigger picture Feeling their research matters Setting realistic goals	Social support Viewing PhD as a process Positive supervisor relationship Self-care

Single = evidenced in a single study; multiple = evidenced across more than one study

## Discussion

This systematic review summarises a heterogeneous research area, with the aim of understanding the mental health of DRs, including possible risk and protective factors. The qualitative and quantitative findings presented here suggest that poor mental health is a pertinent problem facing DRs; stress appears to be a key issue and significantly in excess of that experienced in the general population. Several risk and protective factors at the individual, interpersonal and systemic levels emerged as being important in determining the mental health of DRs. The factors with the strongest evidence-base (i.e. those supported by multiple studies using qualitative and quantitative findings) denote that being female and isolated increases the risk of the mental health problems, whereas seeing the PhD as a process, feeling socially supported, having a positive supervisor relationship and engaging in self-care is protective.

### Results in context

#### Stress

Stress can be defined as (1) the extent to which a stimulus exerts pressure on an individual, and their propensity to bear the load; (2) the duration of the response to an aversive stimuli, from initial alert to exhaustion; or (3) a dynamic (im)balance between the demands and personal resource to manage those demands [91]. The Perceived Stress Scale (PSS) [18, 19] used in our meta-analysis is aligned with the third of these definitions. As elaborated upon within the Transactional Model of Stress [92], stress is conceptualised as a persons’ appraisal of the internal and external demands put upon them, and whether these exceed their available resources. Thus, our results suggest that, when compared to the general population, PhD students experience a greater maladaptive imbalance between their available resources and the demands placed upon them. Stress in itself is not a diagnosable mental health problem, yet chronic stress is a common precipitant to mental health difficulties such as depression and posttraumatic stress disorder [93, 94]. Therefore, interventions should seek to bolster DRs’ resources and limit demands placed on them to minimise the risks associated with acute stress and limit its chronicity.

#### Individual factors

Female DRs were identified as being at particular risk of developing mental health difficulties. This may result from additional hurdles when studying in a male-dominated profession [95–97], and the expectation that in addition to their doctoral studies, females should retain sole or majority responsibility for the domestic and/or caring duties within their family [52, 76]. It may also be that females are more willing to disclose and seek help for mental health difficulties [98]. Nevertheless, the World Health Organisation (WHO) mental health survey results indicate that whilst anxiety and mood disorders are more prevalent amongst females, externalising disorders are more common in males [99]. As the vast majority of studies in this review focussed on internalising problems (e.g. stress, anxiety and depression) [37, 64, 79, 80, 83, 89], this may explain the gender differences found in this review. Further research is needed to explore which perspective, if any, may explain gender gap in our results.

Perhaps unsurprisingly, self-care was associated with reduced mental health problems. The quantitative findings suggest that all types of self-care are likely to be protective of mental health (i.e. physical, emotional, professional and spiritual self-care). Self-care affords DRs the opportunity to take time away from their studies and nurture their non-PhD identities. However, the results from our meta-synthesis suggest that DRs are not attending to their most basic needs much less engaging in self-care behaviours that correspond to psychological and/or self-fulfilment needs [100]. Consequently, an important area for future enquiry will be identifying the barriers preventing DRs from engaging in self-care.

#### Interpersonal factors

Across both quantitative and qualitative studies, interpersonal factors emerged as the most salient correlate of DR mental health. That is, isolation was a risk factor, whereas connectedness to others was a protective factor. There was some variability in how these constructs were conceptualised across studies, i.e. (1) isolation: a lack of social support, having fewer social connections, feeling isolated or being physically separate from others; and (2) social connectedness: multiple group membership, academic relationships or non-academic relationships; but there was no indication that effects varied between concepts. The relationship between isolation and negative health consequences is well-established, for example both physical and mental health problems [101], and even increased mortality [102]. Conversely, social support is associated with reduced stress in the workplace [103, 104]. Reducing isolation is therefore a promising interventional target for improving DRs’ mental health.

The findings regarding isolation are even more alarming when considered alongside the findings from several studies that PhD studies are consistently reported to dominate the lives of DRs, resulting in poor ‘work-life balance’ and losing non-PhD aspects of their identities. The negative impact of having fewer identities [105] can be mitigated by having a strong support network [106], and increasing multiple group memberships [107]. But for DRs, it is perhaps the absence of this social support, combined with identity impoverishment, which can explain the higher than average prevalence of stress found in our meta-analysis.

#### Systemic factors

DRs’ attitudes towards their studies may be a product of top-down systemic issues in academia more broadly. Experiencing mental health problems was reported as being the ‘norm’, but also appeared to be understood as a sign of weakness. The meta-synthesis results suggest that DRs believed their respective universities prioritise academic success over workplace wellbeing and encourage unhealthy working habits. Working in an unsupportive and pressured environment is strongly associated with negative psychological outcomes, including increased depression, anxiety and burnout [108]. The supervisory relationship appeared a particularly important aspect of the workplace environment. The quantitative analysis found a negative correlation between inspirational supervision and mental health problems. Meta-synthetic finding suggested toxic DR-supervisor relationships characterised by powerlessness and neglect, as well as relationships where DRs felt valued and respected—the former of these being associated with poor mental health, and the latter being protective. The association between DR-supervisor relationship characteristics needs to be verified using quantitative methods. Furthermore, DRs’ sense that they needed to exhibit ‘optimum suffering’, which appears to reflect a PhD-specific aspect of a broader academic performativity [109], is an important area for consideration. An accepted narrative around DRs needing to experience a certain level of dis/stress would likely contribute to poor mental health and as an impediment to the uptake and effectiveness of proffered interventions. Although further research is needed, it is apparent that individual interventions alone are not sufficient to improve DR mental health, and that a widespread culture shift is needed in order to prevent the transmission of unhealthy work attitudes and practices.

### Limitations of the literature

Although we found a respectable number of articles in this area, the focus and measures used varied to the extent that typical review analysis procedures could not be used. That is, there was much heterogeneity in terms of how mental health was conceptualised and measured, as well as the range of risk and protective factors explored. Similarly, the quality of the studies was hugely variable. Common flaws amongst the literature include small sample sizes, the use of unvalidated tools and the incomplete reporting of results. Furthermore, for qualitative studies specifically, there appeared to be a focus on breadth instead of depth, particularly in relation to studies using mixed methods.

The generalisability of our findings is limited largely due to the lack of research conducted outside of the US, but also because we limited our review to papers written in English only. The nature of doctoral studies varies in important ways between studies. For example, in Europe, PhD studies usually apply for funding to complete their thesis within 3–4 years and must know their topic of interest at the application stage. Whereas in the US, PhD studies usually take between 5 and 6 years, involve taking classes and completing assignments, and the thesis topic evolves over the course of the PhD. These factors, as well as any differences in the academic culture, are likely to affect the prevalence of mental health problems amongst DRs and the associated risk and protective factors. More research is needed outside of the US.

‘Mental health’ in this review was largely conceptualised as a type of general wellbeing rather than a clinically meaningful construct. None of the studies were ostensibly focused on sampling DRs who were currently experiencing or had previously experienced mental health problems per se, meaning the relevance of the risk, vulnerability and protective factors identified in the meta-synthesis may be more limited in this group. Few studies used clinically meaningful measures. Where clinical measures were used, they captured data on common mental health problems only (i.e. anxiety and depression). Due to these limitations, we are unable to make any assertions about the prevalence of clinical-level mental health problems amongst DRs.

### Limitations of this review

As a result of the heterogeneity in this research area, some of the results presented within this review are based on single studies (e.g. correlation data; see Fig. 5) rather than the amalgamation of several studies (e.g. meta-analysis/synthesis). To aid clarity when interpreting the results of this review, we have (Table 3) summarised the volume of evidence supporting risk and protective factors. Moreover, due to the small number of studies eligible for inclusion in this review, we were unable to test whether any of our findings are related to the study characteristics (e.g. year of publication, country of origin, methodology).

We were able to conduct three meta-analyses, one of which aimed to calculate the between-group effect size on the PSS [18, 19] between DRs and normative population data. Comparing these data allowed us to draw some initial conclusions about the prevalence of stress amongst DRs, yet we were unable to control for other group differences which might moderate stress levels. For example, the population data was from people in the United States (US) in 1 year, whereas the DR data was multi-national at a variety of time points; and self-reported stress levels may vary with nationality [110] or by generation [111, 112]. Moreover, two of the three meta-analyses showed significant heterogeneity. This heterogeneity could be explained by differences in the sample characteristics (e.g. demographics, country of origin), doctoral programme characteristics (e.g. area of study, funding status, duration of course) or research characteristics (e.g. study design, questionnaires used). However, due to the small number of studies included in these meta-analyses, we were unable to test any of these hypotheses and are therefore unable to determine the cause of this heterogeneity. As more research is conducted on the mental health of DRs, we will be able to conduct larger and more robust meta-analyses that have sufficient power and variance to statistically explore the causes of this heterogeneity. At present, our findings should be interpreted in light of this limitation.

### Practice recommendations

Although further research is clearly needed, we assert that this review has identified sufficient evidence in support of several risk and protective factors to the extent that they could inform prevention and intervention strategies. Several studies have evidenced that isolation is toxic for DRs, and that social support can protect against poor mental health. Initiatives that provide DRs with the opportunity to network and socialise both in and outside of their studies are likely to be beneficial. Moreover, there is support for psychoeducation programmes that introduce DRs to a variety of self-care strategies, allow them to find the strategies that work for them and encourage DRs to make time to regularly enact their chosen strategies. Finally, the supervisory relationship was identified as an important correlate of DR mental health. Positive supervision was characterised as inspirational and inclusive, whereas negative supervision productised DRs or neglected them altogether. Supervisor training programmes should be reviewed in light of these findings to inform how institutions shape supervisory practices. Moreover, the initial findings reported here evidence a culture of normalising and even celebrating suffering in academia. It is imperative therefore that efforts to improve and protect the mental health of DRs are endorsed by the whole institution.

### Research recommendations

First, we encourage further large-scale mental health prevalence studies that include a non-PhD comparison group and use validated clinical tools. None of the existing studies focused on the presence of serious mental health problems—this should be a priority for future studies in this area. Mixed-methods explorations of the experiences of DRs who have mental health problems, including serious problems, and in accessing mental health support services would be a welcome addition to the literature. More qualitative studies involving in-depth data collection, for example interview and focus group techniques, would be useful in further supplementing findings from qualitative surveys. Our review highlights a need for better communication and collaboration amongst researchers in this field with the goal of creating a level of consistency across studies to strengthen any future reviews on this subject.

## Conclusions

The results from this systematic review, meta-analysis and meta-synthesis suggest that DRs reported greater levels of stress than the general population. Research regarding the risk and protective factors associated with the mental health of DRs is heterogenous and disparate. Based on available evidence, robust risk factors appear to include being isolated and being female, and robust protective factors include social support, viewing the PhD as a process, a positive DR-supervisor relationship and engaging in self-care. Further high-quality, controlled research is needed before any firm statements can be made regarding the prevalence of clinically relevant mental health problems in this population.

## Supplementary information

**Additional file 1.**

## Data Availability

The datasets used and/or analysed during the current study are available from the corresponding author on reasonable request.

## Abbreviations

CI: Confidence intervals
DR: Doctoral researchers
HESA: Higher Education Statistics Agency
M: Mean
PSS: Perceived Stress Scale
SD: Standard deviation
UK: United Kingdom
US: United States
