# Supplementary material for: Understanding the mental health of doctoral researchers: a mixed methods systematic review with meta-analysis and meta-synthesis
Source: Syst Rev. 2020 Aug 26;9:197. doi: 10.1186/s13643-020-01443-1 (PMC7450565; doi:10.1186/s13643-020-01443-1)
Supplement: Supplementary file 1 — Additional file 1. [file 13643_2020_1443_MOESM1_ESM.docx]

**Understanding the mental health of Doctoral Researchers: A mixed methods systematic review with meta-analysis and meta-synthesis.**

# Literature Search Strategy:

## Databases searched:

- AMED;
- BNI;
- CINAHL;
- Embase;
- HBE;
- HMIC;
- Medline;
- PsycInfo;
- PubMed;
- Scopus;
- Web of Science.

Additional paper were added by searching the reference list of all eligible papers.

## Search terms:

(“PhD student*” OR “doctoral student*” OR “postgraduate research student*” OR “doctoral research student*” OR “research student*” OR “doctoral researcher” OR “Doctoral Researcher” OR “doctoral level” OR “PhD candidate*”)

AND

(“mental health” OR “mental disorder” OR “mental illness” OR “psychiatric illness” OR “psychiatric problems” OR “psychiatric symptom*” OR “psychological well*being” OR “distress” OR “psychopathology” OR “depress*” OR “anxiety” OR “mood disorder*” OR “affective disorder” OR “panic disorder” OR “OCD” OR “obsessive-compulsive” OR “obsessive compulsive” OR “post-traumatic” OR “social phobia” OR “phobia” OR “phobic” OR “GAD” OR “PTSD” OR “SAD” OR “psychosis” OR “psychotic” OR “delus*” OR “hallucinat*” OR “mania” OR “manic” OR “personality disorder*” OR “bipolar” OR “schizo*” OR “anorexia nervosa” OR “bulimia nervosa” OR “eating disorder” OR “binge eating” OR “insomnia”)
